# Fostering creativity in engineering education: The CEDE project and the design of a European e-learning platform

**DOI:** 10.12688/openreseurope.23240.2

**Published:** 2026-06-23

**Authors:** Cristina Prado-Acebo, Ana Ares-Pernas, Zuzana Palková, Hatice Uzuğ, Ramazan Kocatürk, Athanasios Angeioplastis, Alkiviadis Tsimpiris, Rossella Nocera, Antonio Sandoli

**Affiliations:** 1 Universidade da Coruña, Campus Industrial de Ferrol, Escuela Universitaria de Diseño Industrial, Ferrol, A Coruña, 15403, Spain; 2 Slovak University of Agriculture in Nitra, Nitra, 949 76, Slovakia; 3 Institute of Technology and Business in České Budějovice, České Budějovice, 370 01, Slovakia; 4 Kocatürk Consulting, PınarMah, Kocaeli, 74217, Turkey; 5 International Hellenic University, Informatics and Telecommunications Engineering, Serres, 62124, Greece; 6 University of Molise, Department of Biosciences and Territory, Campobasso, 80266, Italy

**Keywords:** Engineering education; Creativity; Critical thinking, Design thinking; Digital competences; Problem solving; Soft skills; E-learning.

## Abstract

**Background:**

Engineering plays a central role in today’s technology-driven society, requiring professionals not only with strong technical knowledge but also with creative, critical and digital competences. However, creativity is still insufficiently integrated into many engineering curricula. The Erasmus+ project CEDE – Creative Engineering addresses this gap by promoting innovative educational approaches aimed at enhanced creativity and related transversal skills in engineering higher education.

**Methods:**

The project follows a collaborative, international methodology involving five European countries (Spain, Italy, Greece, Turkey and Slovakia). First, a comparative analysis was carried out to examine how creativity is currently addressed in engineering degrees programmes at partner universities and national contexts. Based on this analysis, a common complementary module was designed, structured into five thematic areas: critical thinking, design thinking, digital problem solving, digitalization and artificial intelligence, and soft skills. Each module includes specific learning outcomes, teaching methodologies and assessment tools aimed at supporting the development of creative competences. An e-learning platform was developed to deliver the content. Feedback was collected through round tables with students, academic staff, university staff, professionals and company representatives, and used to iteratively refine the modules.

**Results:**

The comparative report identified variability in the treatment of creative competences across institutions, as well as opportunities for their more explicit integration within engineering curricula. The resulting common module provides a flexible framework that can be adapted to different engineering programmes. Stakeholder feedback indicates the perceived relevance, usability and pedagogical value of the modules and the e-learning platform, and contributed to their iterative refinement.

**Conclusions:**

The CEDE project demonstrates the feasibility and value of systematically integrating creativity-related competences into engineering education through participatory and interdisciplinary approaches. This approach not only fosters creativity but also prepares engineers to tackle complex, interdisciplinary challenges in a rapidly evolving technological landscape.

## Plain Language summary

This article presents an European project called CEDE (Creative Engineering), which aims to improve how engineering is taught at university. The main idea is that today’s engineers need more than technical knowledge: they also need creativity, critical thinking, teamwork, communication skills, and the ability to use digital tools responsibly.

To address this, universities from five European countries (Spain, Italy, Greece, Turkey and Slovakia) worked together to design a shared online course that can be easily added to different engineering degrees. The course is organised into five areas: critical thinking, design thinking, digital problem solving, digitalization and artificial intelligence, and soft skills. An e-learning platform was developed to deliver the content.

Students, teachers, and companies took part in evaluating the course and confirmed that it is useful, practical, and relevant to real professional needs. The project shows that creativity can be taught in a structured way and that doing so helps to prepare engineers to face today’s complex technological and social challenges.

## 1. Introduction

Engineering plays a central role in today’s technology-driven society, shaping technological, industrial and social development. For this reason, professionals, not only with strong technical knowledge but also with creative, critical and digital competences, are required ( Pusca & Northwood, 2019). Engineers create the objects and systems we use every day; they design systems, products and infrastructures that respond to increasingly complex global challenges, including digital transformation, sustainability, and rapid technological change. Therefore, they must be able to see things in a creative way, to recognize needs, and new ideas by thinking “out of the box” to solve problems from a creative point of view.

In this context, engineering practice requires not only solid technical knowledge but also a broad set of transversal competences, such as creativity, critical thinking, problem solving, digital competence and communication skills. These competences are essential for addressing problems, generating innovative solutions and adapting to evolving professional environments ( Kuimova et al., 2017; Peroni, 2024). So, it is very important to teach and encourage creativity in engineering degrees and to develop programs that train on these competences.

However, creativity is still insufficiently integrated into many engineering curricula. The Erasmus+ project CEDE – Creative Engineering addresses this gap by promoting innovative educational approaches aimed to enhanced creativity and related transversal skills in engineering higher education.

Despite this growing demand, traditional engineering education has often prioritized technical content and analytical problem solving, leaving limited space for the systematic development of creative and transversal skills ( Lawson, 2009). Creativity is frequently perceived as an innate ability or as a competence more closely associated with artistic disciplines rather than with engineering ( Brown, 2008). As a result, creative approaches are often marginalized within engineering curricula or addressed in an implicit and fragmented manner. This gap between the competences required by society and industry ( Florida, 2010; Beckman and Barry, 2007, p. 25) and those fostered in higher education has been widely recognized as a challenge for engineering education.

In recent years, educational research has highlighted the importance of integrating creativity into engineering programs ( Kirillov et al., 2015) through active learning methodologies, interdisciplinary approaches and student-centered pedagogies. Approaches such as design thinking, project-based learning and problem-based learning have shown potential to enhance students’ creative capacities, critical thinking and engagement. At the same time, the rapid development of digital technologies has opened new opportunities for the design of flexible learning environments, including e-learning platforms that support collaborative, self-directed and experiential learning. However, the implementation of such approaches remains uneven across institutions and countries, particularly in non-core campuses and smaller universities.

Within this context, European cooperation projects offer a valuable framework for addressing common educational challenges and promoting innovation in higher education. Erasmus+ projects foster collaboration among institutions from different countries, enabling the exchange of experiences, the comparison of educational practices and the co-creation of shared educational resources. The CEDE – Creative Engineering project (2023–2025) was conceived in response to the need to strengthen creativity and related transversal skills in engineering education through a structured, collaborative and international approach.

The CEDE project involves five higher education institutions from non-core campuses in five European countries (Spain, Italy, Greece, Turkey and Slovakia, the leader of the project), bringing together educators from different engineering disciplines and institutional contexts. A distinctive feature of the project is its focus on non-core campuses, which often face additional challenges in terms of resources, visibility and curricular innovation. By engaging partners from diverse geographical and educational contexts, the project seeks to develop a flexible framework that can be adapted to different engineering programs while addressing shared learning objectives.

The main objective of the CEDE project is to contribute to the development of creative skills in engineering students, together with other key competences such as critical thinking, problem solving, digital thinking and soft skills. To achieve this objective, the project aims to design and implement a common complementary module that can be integrated into existing engineering curricula at an early stage. This module is delivered through an e-learning platform and is structured into five thematic areas, each coordinated by one of the partner countries: critical thinking, design thinking, digital problem solving, digital thinking and artificial intelligence, and soft skills. The modular design allows for both comprehensive implementation and selective adoption, depending on the specific needs of each institution. Thus, by developing creative engineering modules on an e-learning platform, the aim is to improve the professional profiles of future engineers.

An additional objective of the project is to enhance the attractiveness of engineering degrees and to support a deeper understanding of engineering concepts by promoting creative and reflective learning processes ( McIntosh, 2010; Mentzer et al., 2015, p. 428). By encouraging students to think “outside the box”, recognize needs and explore multiple solution paths ( Dorst & Cross, 2001, p. 435), the project seeks to align engineering education more closely with real-world professional practice. Furthermore, the involvement of multiple stakeholders, including students, teachers, university staff and representatives from industry, aims to ensure the relevance and applicability of the developed modules.

This research article presents the design, implementation and initial outcomes of the CEDE project, with a particular focus on the development of the European e-learning platform and the associated creative engineering modules. The study adopts a research-oriented perspective, combining comparative analysis, curricula design and participatory evaluation methods. The paper is structured as follows. First, the Methods section describes the different methodologies used on each stage of the project. The Results section presents the main findings of the comparative report and provides a concise overview of each module developed by the partner countries. Finally, the Conclusions and Discussion section reflects on the implications of the project for engineering education and outlines future directions for the integration and dissemination of creativity-focused educational initiatives.

## 2. Methods

### 2.1 Research design

The CEDE project adopts a qualitative and design-based research (DBR) approach ( Laursen & Haase, 2019) aimed at the development, implementation and iterative refinement of an educational intervention in engineering higher education. Design-based research is particularly suitable for this study, as it enables the systematic integration of analysis, design, and evaluation processes in real educational settings.

The methodological framework is structured into several interrelated phases. First, a comparative curriculum analysis was conducted across partner institutions to identify how creativity-related competences and transversal skills are currently addressed in engineering programmes. Second, based on these findings, an instructional design phase was carried out, involving the co-creation of educational modules and the definition of their pedagogical structure. Third, a participatory evaluation phase was implemented through stakeholder engagement activities, including round tables with students, academic staff, professionals, and company representatives. Finally, these inputs informed the development and refinement of an e-learning platform designed to support the implementation of the modules.

Data sources included curriculum documents, a shared analysis template used by all partners, records and summaries from stakeholders round tables, and feedback collected during the developments and testing of the modules and platform. These data were analysed qualitatively, with a focus on identifying recurring themes, needs, and patterns that could inform the iterative design process.

The project was implemented between 2023 and 2025 within the framework of the Erasmus+ programme and involved five higher education institutions from Spain, Italy, Greece, Turkey and Slovakia. Participants including teachers from different engineering disciplines, educational researchers, institutional staff, and external stakeholders involved in different phases of the study.

### 2.2 Comparative analysis of engineering curricula

The first methodological phase consisted of a comparative curriculum analysis aimed at examining how creativity and related transversal competences are addressed in existing engineering degree programmes at the partner universities. Each institution analysed a selection of engineering programmes at institutional and national level, focusing on the presence of learning outcomes, courses, and learning activities explicitly or implicitly related to creativity, critical thinking, problem solving, digital competences and soft skills.

To ensure comparability across countries, all partners agreed on a common analysis template, which includes criteria related to (i) the explicit inclusion of transversal competences in programme learning outcomes, (ii) the integration of these competences within courses and teaching practices, and (iii) the assessment approaches used. This template guided the systematic collection of data across institutions.

The collected data included qualitative descriptions of curricular structures, teaching methodologies and assessment practices, as well as contextual information regarding institutional frameworks, constraints and opportunities. The analysis followed a collaborative and iterative process, in which partner institutions contributed their findings to a shared dataset, enabling cross-case comparison and the identification of recurring patterns.

The results of this phase were synthesised into a comparative report, which served as the empirical basis for the subsequent instructional design phase, informing both the definition of the module structure and the selection of key competences to be addressed.

### 2.3 Design of the common creative engineering module

Based on the findings of the comparative analysis, a common complementary module was designed with the objective of fostering creativity and transversal skills in engineering students. The module was conceived as a flexible and adaptable structure that could be integrated into different engineering programmes at an early stage of the curriculum.

The module is organised into five thematic areas, each coordinated and developed by one partner country:

1. Critical Thinking: Introduction to Creative Engineering (Italy)
2. Design Thinking: Creative Engineering Methods (Spain)
3. Digital Thinking: Problem Solving (Turkey)
4. Digital Thinking: Digitalisation and Artificial Intelligence (Greece)
5. Soft Skills (Slovakia)

For each thematic area, the responsible partner designed the learning content, teaching methodology and assessment strategy. A competency-based approach was adopted, with clearly defined learning outcomes aligned with creativity-related competences. Regular consortium meetings were held to ensure coherence across modules and to harmonise pedagogical approaches.

### 2.4 Development of the E-learning platform

To support the delivery of the common module, an e-learning platform was developed as a central element of the project. The platform was designed to facilitate flexible access to learning materials, interactive activities and assessment tools. It supports self-directed learning, collaborative tasks and project-based activities, allowing students to engage with the content at their own pace.

The platform hosts the five modules and provides a structured learning pathway, including theoretical content, practical exercises, tests, case studies and project work. Particular attention was paid to usability, accessibility and adaptability to different institutional contexts. The platform also integrates assessment and monitoring tools to track students’ progress in the development of creative competences.

### 2.5 Assessment tools and monitoring of creative competences

To evaluate and monitor the development of creative competences, a total of fifteen assessment tools were selected and designed within the project, with three tools associated with each module. These tools include a combination of formative and summative assessment methods, such as knowledge tests, portfolios, project work, presentations, self-assessment and peer assessment activities.

The assessment framework aims to capture multiple dimensions of creativity and transversal skills, including ideation, problem analysis, solution development, collaboration and communication. The design of the assessment tools was aligned with the learning outcomes of each module and adapted to the online learning environment.

### 2.6 Round table, stakeholder feedback and iterative improvement

A key methodological component of the project was the organisation of round tables aimed at evaluating the e-learning platform and its contents. These activities were conducted across the five participant countries and involved a diverse group of stakeholders, including students, academic staff, university staff, professionals and representatives from companies in the engineering sector.

During the round tables, participants were invited to explore the platform, review the modules and provide structured feedback on aspects such as relevance, clarity, usability, pedagogical value and alignment with professional needs. Data collection combined guided group discussions and questionnaire-based input, allowing both open qualitative insights and structured responses to be gathered.

The analysis followed a collaborative cross-institutional process, in which partner institutions synthesised their results and identified common patterns across contexts. While some variability was observed due to institutional and national differences, the feedback showed consistent trends regarding the perceived relevance of transversal competences and the value of integrating creativity-oriented approaches into engineering education.

The results were used to iteratively refine the modules, assessment tools, and platform functionalities. In line with the design-based research approach, this feedback loop supported the progressive adjustment of the educational intervention to stakeholder needs.

For illustrative purposes, selected results are presented in more detail for the University of A Coruña case, while the overall findings reflect shared patterns identified across partner institutions. This is acknowledged as a limitation in terms of the level of detail available for each context.

### 2.7 Dissemination

The final methodological phase focused on the dissemination of the project results and the iterative improvement of the developed materials. Dissemination activities included multiplier events, participation in congress, presentations at academic and professional forums, media, social nets and the sharing of resources within the partner institutions.

## 3. Results

### 3.1 Results of the comparative analysis of engineering curricula

Engineering curricula varies significantly across different institutions and countries, influenced by factors such as industrial needs, educational policies, and cultural priorities ( Arnold, 2016). A comparative analysis of the curriculum reveals distinct approaches to teaching and skill acquisition in engineering disciplines. In this project the key aspects of engineering curricula from various regions have been outlined before defining the E-learning platform.

From a general standpoint, key differences among the curricula were analysed regarding a) Duration and Structure, b) Emphasis on Practical Experience, c) Project-Based Curriculum.

Moreover, all the aspects related to the course organizations in various countries involved in the CEDE project – such as duration, focus area, project-based learning, interdisciplinarity and accreditation – were analysed and compares.

The comparisons showed a marked trend toward incorporating more digital skills and sustainability into engineering curricula. Universities worldwide are beginning to integrate topics like artificial intelligence, renewable energy systems, and data analytics, reflecting industry demands and global challenges.

The comparative curriculum in engineering courses highlights the diversity in educational strategies and priorities worldwide. Understanding these differences can help prospective students choose the program that aligns best with their career goals and interests, while also providing insights into how different regions prepare their engineering graduates for the global workforce.

### 3.2 Results of the E-learning platform and the modules developed by each partner country

The modules developed by each partner institution constitute one of the main outcomes of the CEDE project. This section presents their structure and pedagogical orientation, with a focus on how they operationalise the competence framework identified through the comparative curriculum analysis and respond to the needs highlighted during the stakeholder feedback phase.

While the level of detail provided for each module may appear extensive, this reflects the project’s design-based approach, in which educational interventions are iteratively developed, documented, and refined. The descriptions therefore aim not only to present the modules, but also to illustrate how creativity and transversal competences are embedded into engineering education through concrete pedagogical design.

The modules share a common structure—combining conceptual foundations, applied methodologies, and project-based learning—while allowing adaptation to specific disciplinary and institutional contexts. The following subsections highlight the main features of each module and their contribution to the overall CEDE framework.

*3.2.1 Critical Thinking: Introduction to Creative Engineering (Italy)*

Creativity is often associated with the arts, but creativity is needed everywhere, and it plays a crucial role in engineering as well. Engineers must always find solution to real world problems. They need to think of creative ways to come up with solutions. Creativity allows engineers to deal with new problems and new applications combined with existing and traditional methods and techniques.

Creative engineering is the place where innovation meets practicality, and imagination drives progress. In this dynamic field, engineers blend their technical expertise with a dash of creativity to solve complex problems, design ground-breaking products, and shape the future of technology. It transcends traditional boundaries, encompassing diverse disciplines such as mechanical, electrical, software, and aerospace engineering, among others. It thrives on the intersection of art and science, encouraging engineers to think outside the box and push the limits of what’s possible.

In summary, creativity is a vital component of engineering that fuels innovation, drives problem-solving, and enables engineers to develop novel and impactful solutions to complex challenges. By embracing creativity, engineers can unlock new possibilities, drive progress, and make a positive impact on society and the world at large.

The aim of the competence unit is to develop a comprehensive set of skills that enable engineers to have a comprehensive vision of the basic concepts of the creative engineering, allowing them to acquire the basic competences to face the subsequent modules. It encompasses a wide range of topics, spanning from fundamental principles to advanced techniques.

The first part of the module involves “Defining creativity”. It is a nuanced task, as it encompasses various aspects and can manifest differently depending on the context. However, at its core, creativity can be understood as the ability to generate novel and valuable ideas, solutions, or outcomes.

The unit covers various problem-solving techniques and strategies relevant to creative engineering, as described in the following:

1. Novelty: Generating ideas, solutions, or outcomes that are original and new, ranging from entirely unprecedented concepts to innovative combinations of existing elements.
2. Value: Producing ideas or solutions that are meaningful, useful, or impactful in some way. The value of creative outputs can be subjective and context-dependent.
3. Problem-solving: Approaching challenges with an open mind, exploring multiple perspectives, and generating unconventional solutions.
4. Flexibility: Flexibility in thinking, willing to entertain diverse ideas and perspectives. They are not bound by conventional or rigid thinking patterns and are open to exploring alternative approaches.
5. Originality: Building upon existing ideas or concepts, it also requires a degree of originality. Creative individuals bring their unique insights, experiences, and perspectives to the creative process, resulting in outputs that reflect their individuality.
6. Expression: Creativity it encompasses artistic expression and innovation in areas such as literature, music, art, and design. Creative expression allows individuals to communicate emotions, ideas, and experiences in novel and engaging ways.
7. Process-oriented: Creativity is often viewed as a process rather than a fixed trait. It involves engaging in activities such as brainstorming, experimentation, iteration, and reflection to generate and refine creative ideas and solutions.
8. Context-dependence: The perception of creativity can vary depending on cultural, social, and disciplinary contexts. What is considered creative in one context may not be perceived as such in another. Additionally, the criteria for evaluating creativity may differ across domains.

The core unit number two engages the “Creativity in engineering”. The creative process involves several steps. The first level includes the definition of the question or the problem. The process goes further involving all ideas and concepts associated to the problem. By following these steps described in the following and leveraging creative thinking techniques, engineers can navigate the complexities of engineering challenges and develop innovative solutions that address the needs of society, industry, and the environment.

The unit covers various problem-solving techniques and strategies relevant to creative engineering, as described in the following:

1. Identifying the Problem: The first step in the creative process is to clearly define the problem or challenge that needs to be addressed. This involves understanding the requirements, constraints, and objectives of the project, as well as gathering relevant information and data.
2. Research and Exploration: Enable the engineers capable to conduct research and explore existing solutions, technologies, and methodologies related to the problem at hand. This helps them gain insights, identify best practices, and understand the current state of the art in the field.
3. Brainstorming and Idea Generation: Brainstorming sessions are conducted to generate a wide range of ideas, concepts, and potential solutions. Engineers leverage creative thinking techniques such as free association, mind mapping, and lateral thinking to explore diverse possibilities and generate novel insights.
4. Concept Development: Once a pool of ideas has been generated, engineers evaluate and refine them to develop more concrete concepts and proposals. This may involve sketching, prototyping, or modeling to visualize and communicate ideas effectively.
5. Problem-solving: Enable the engineers to approach to problems from different angles, consider unconventional solutions, and develop innovative strategies to address them effectively.
6. Innovative Design: Engineers must devise innovative solutions that meet specific requirements while considering factors such as functionality, efficiency, safety, and cost-effectiveness. Creativity allows engineers to explore novel concepts, integrate new technologies, and push the limits of design possibilities.
7. Optimization and Efficiency: Creativity as a tool for optimizing existing processes, systems, and products. Engineers continually seek ways to improve efficiency, enhance performance, and reduce waste through creative problem-solving and innovation. This may involve redesigning components, reconfiguring processes, or implementing new technologies.
8. Refinement and Optimization: The design is refined and optimized based on the feedback obtained during prototyping and testing. Engineers iterate on the design, making adjustments, improvements, and optimizations to enhance performance, functionality, and usability.
9. Interdisciplinary Collaboration: Many engineering projects require collaboration across multiple disciplines, such as mechanical, electrical, software, and materials engineering. Creativity enables engineers to communicate effectively across disciplinary boundaries, leverage diverse expertise, and integrate different perspectives to develop holistic solutions.
10. Risk Management: Creativity is integral to managing risks in engineering projects. Engineers must anticipate potential challenges, identify vulnerabilities, and develop creative strategies to mitigate risks effectively. This may involve contingency planning, scenario analysis, and innovative approaches to ensure project success.
11. Sustainability and Environmental Responsibility: In today’s world, sustainability and environmental considerations are paramount in engineering design and decision-making. Creativity enables engineers to develop eco-friendly solutions, minimize environmental impact, and promote sustainable practices through innovative design, materials selection, and energy-efficient technologies.
12. Continuous Improvement and Innovation: Creativity drives continuous improvement and innovation in engineering. Engineers are constantly seeking ways to push the boundaries of what is possible, develop new technologies, and pioneer groundbreaking solutions to address evolving needs and challenges.
13. Human-Centered Design: Creativity is essential for human-centered design, which focuses on creating products, systems, and experiences that prioritize the needs, preferences, and capabilities of end-users. Engineers leverage creative thinking to empathize with users, anticipate their requirements, and design solutions that enhance usability, accessibility, and user satisfaction.
14. Reflection and Learning: After the completion of the project, engineers will reflect on the creative process, evaluate the outcomes, and identify lessons learned. This reflection helps them gain insights, improve their skills, and apply their experiences to future projects.

The last part of the module involves the “Creation of a project”, based on team-working to bring diversity of background and expertise together. In the realm of Creative Engineering, the creation of a project begins with a unique blend of innovation and practical problem-solving. Engineers leverage their technical knowledge and artistic vision to develop solutions that address specific needs or challenges. This process often starts with brainstorming sessions where diverse ideas are generated, followed by concept development and prototyping. Teams incorporate feedback and iteration to refine their designs, ensuring both functionality and aesthetic appeal. Throughout the project, collaboration across disciplines—such as design, engineering, and user experience—is crucial, fostering an environment where creativity thrives. Ultimately, the successful realization of a project in Creative Engineering not only showcases technical expertise but also inspires new ways to think about and engage with technology in everyday life.

This final step is organised in different sub-fields:

1. Introduction to Creative Engineering: Overview of Creative Engineering, with definitions and significance. Historical Context about sey developments in engineering and design. Case Studies: Examples of successful creative engineering projects.
2. Ideation and Brainstorming: Techniques for Ideation such as mind mapping, SCAMPER, and role-playing. Collaborative Brainstorming creating group dynamics and idea generation. Evaluating ideas through criteria for assessing feasibility and innovation.
3. Design Thinking Process: Understanding user needs, conducting interviews and surveys and defining problem statements. Prototyping Techniques: Low fidelity vs. high-fidelity prototypes.
4. Technical Skills Development. It involves basic engineering principles such as introduction to materials and methodologies and development of experiments. It is required the use of software tools for students, i.e., overview of CAD, simulation software, and project management tools.
5. Project Management in Creative Engineering. It is aimed at developing planning and organization, setting goals, timelines, and budgeting. The team work dynamically, defining roles and collaboration in project teams, iterative design and feedback and the importance of testing and refining ideas.
6. Presentation and Communication Skills. It involves the development of effective communication techniques for presenting ideas clearly and the visual design using visuals to enhance presentations and projects. Moreover, storytelling in engineering is learned, crafting compelling narratives for project pitches.
7. Sustainability and ethics in engineering: sustainable design practices. Here are learned the principles of eco-friendly engineering, ethical considerations about the responsibility and impact of engineering solutions, and future trends in emerging technologies and their potential effects on society.
8. Final Presentation: Showcasing the project to peers and instructors. These submodules can guide students through various key aspects of Creative Engineering, helping them build a comprehensive skill set.

This module illustrates how creativity can be explicitly structured within engineering education, addressing gaps identified in the initial curriculum analysis and aligning with the competence framework proposed by the CEDE project.

*3.2.2 Design Thinking: Creative Engineering Methods (Spain)*

The aim of this competence unit and its associated learning outcomes is to equip students with the necessary skills and knowledge to manage design thinking and creative engineering methods in their future work as engineers, to work collaboratively in multidisciplinary teams and to apply creativity in the design of new products and services. The module addresses the need to connect technical knowledge with innovative thinking processes, enabling students to approach engineering challenges from a user-centred, iterative and solution-oriented perspective.

This module was designed in response to the results of the comparative analysis, which revealed that while problem-solving is a core component of engineering education, explicit training in creative methodologies and design thinking is often limited or fragmented. The Spanish partner therefore developed a module that integrates creativity directly into engineering practice, positioning design thinking not as an auxiliary skill ( Cross, 1982, p. 223; Buchanan, 1992) but as a structured methodology applicable to real engineering contexts.

The module is organised into three interrelated sections that progressively guide students from conceptual understanding to practical application, ensuring a coherent learning progression from theory to practice. This structure reflects the underlying philosophy of design thinking as a holistic and iterative approach to problem solving in engineering contexts.

The first section focuses on the definition and theoretical foundations of design thinking ( Rowe, 1987; Dorst, 2011, p. 521; Laursen & Haase, 2019; Mosely et al., 2018, p. 177). Students are introduced to the origins, principles and relevance of design thinking within engineering and innovation processes. Emphasis is placed on understanding design thinking as an iterative and non-linear approach ( Luchs et al., 2016, p. 8; Luka, 2020, p. 65) that integrates empathy, problem definition, ideation, prototyping and evaluation. Through this introduction, students develop awareness of the importance of understanding user needs, contextual constraints and societal impact when designing engineering solutions.

Design thinking is presented as a human-centred approach to innovation that integrates analytical and creative processes to address complex and ill-defined problems ( Brown, 2008; Razzouk & Shute, 2012, p. 330). Within the module, design thinking is defined as a structured methodology that combines empathy, creativity and rationality to meet user needs while respecting technical, economic and contextual constraints. Students are introduced to design thinking as an approach that goes beyond traditional linear problem-solving models, emphasising iteration, feedback and continuous refinement.

Particular attention is given to the design thinking process, commonly described through five interconnected phases: empathy, problem definition, ideation, prototyping and testing ( Henriksen et al., 2017). In addition, students are introduced to different design thinking methodologies and frameworks, each with its own structure and emphasis, allowing them to develop a global overview of the process ( Brown and Wyatt, 2010; Tschimmel, 2012; Lawson, 2004). This comparative perspective enables students to adapt and tailor the design thinking approach to the specific needs of their project, their team composition and the context in which the engineering challenge is situated ( Cross, 2001, p. 49–50; Koro & Tanggaard, 2022). Students explore each phase in detail, understanding that these stages are not sequential steps but rather flexible and iterative components of a dynamic process.

In the empathy phase, students learn techniques for understanding users, stakeholders and contexts, such as observation, interviews and user profiling. This phase is used to highlight the importance of incorporating social, ethical and environmental considerations into engineering design.

The problem definition phase focuses on synthesising insights gathered during empathy activities to formulate clear and meaningful problem statements. Students are trained to reframe technical challenges from a user-centred perspective, learning how the formulation of the problem directly influences the quality and creativity of potential solutions. This phase encourages critical thinking and reflection, helping students move beyond predefined problem statements.

In the ideation phase, students are introduced to divergent thinking techniques aimed at generating a wide range of possible solutions. Creativity is framed not as spontaneous inspiration but as a skill that can be developed through structured methods. Emphasis is placed on creating an open and collaborative environment that encourages risk-taking and the suspension of premature judgement. The module highlights the value of quantity, diversity and originality of ideas as a foundation for innovation in engineering design.

The prototyping and testing phases are presented as essential mechanisms for learning through making and experimentation. Students learn that prototypes do not need to be fully functional technical solutions but can take the form of sketches, mock-ups, simulations or conceptual models. These phases reinforce the idea that failure and iteration are integral parts of the design process, fostering resilience and adaptability—key competences for future engineers.

The second section of the module concentrates on creative engineering methods and tools that support and operationalise the design thinking process. This section provides students with a repertoire of structured techniques that can be systematically applied within engineering projects to enhance creativity and innovation ( Engel, 2018). Methods for idea generation, such as brainstorming and other creative problem-solving techniques ( Goria, 2017; Hatcher et al., 2018; Liedtka & Ogilvie, 2011; Wade & Piccinini, 2020), are introduced and practised within a controlled framework to ensure productive collaboration ( Dunne and Martin, 2006) and effective outcomes.

Beyond idea generation, the module addresses tools for concept development, evaluation, selection and refinement. Students are trained to analyse and compare alternative solutions based on technical feasibility, user needs and contextual constraints. These methods are contextualised within engineering scenarios to ensure their applicability and relevance, allowing students to understand how creativity can be systematically integrated into technical design and rigorous engineering decision-making processes, rather than relying solely on intuition, filling the perceived gap between creativity and technical rationality.

All creative engineering methods are contextualised through examples and practical exercises drawn from engineering scenarios. This contextualisation ensures that students perceive creativity as an integral part of engineering practice rather than as an abstract or peripheral competence. By applying these methods to discipline-specific problems, students develop confidence in using creative approaches within their future professional environments.

The third section of the module is dedicated to the development of a technical project, which constitutes the central learning activity and the main evidence of competence acquisition. This project-based component requires students to work collaboratively in teams to address a real or realistic engineering challenge by applying design thinking principles and creative engineering methods. The project serves as a synthesis of the knowledge, skills and attitudes developed throughout the module.

During the project, students are required to define the problem, analyse technical and contextual constraints, generate and evaluate alternative solutions and justify their design decisions. The emphasis is placed not only on the final solution but also on the design process itself. Students are encouraged to document their decisions, reflect on iterations and demonstrate how user needs and creativity have informed their engineering choices.

This section fosters a range of transversal competences in addition to creativity, including teamwork, communication, critical thinking and decision-making skills, reflecting the complexity of professional engineering practice. The collaborative nature of the project shows professional engineering practice, where solutions are typically developed in multidisciplinary teams and require negotiation, coordination and shared responsibility. By engaging in this project-based experience, students gain insight into the complexity of real-world engineering challenges and the value of creative, user-centred approaches.

From a methodological perspective, the module adopts an active and student-centred learning approach. Teaching strategies combine short theoretical inputs with practical activities, collaborative work and guided reflection. Project-based learning serves as the core methodology, supported by complementary techniques such as brainstorming sessions, group discussions, debates and flipped classroom elements. This pedagogical approach encourages students to take an active role in their learning process, promoting engagement, motivation and deeper understanding.

The e-learning platform plays a key role in the implementation of the module. All learning materials, activities and assessment tasks are delivered through the platform, enabling flexible access and supporting both synchronous and asynchronous learning. The digital environment facilitates collaboration among students, provides spaces for sharing ideas and project outcomes, and supports continuous interaction between students and instructors. The use of an online platform also allows the module to be easily integrated into different engineering programmes and institutional contexts.

Assessment in the Design Thinking module is closely aligned with the targeted learning outcomes and creative competences. Three complementary assessment tools are used to capture different dimensions of student learning. First, a test associated with the theoretical content evaluates students’ understanding of design thinking concepts and creative engineering methods. Second, a portfolio corresponding to the technical project documents the design process, including problem definition, ideation, decision-making and final outcomes. This portfolio-based assessment allows for the evaluation of creativity as a process rather than solely as a final product. Third, students present their projects through an online presentation, which assesses communication skills, teamwork and the ability to justify and reflect on creative solutions.

To ensure transparency and consistency, assessment criteria are defined through detailed rubrics that are shared with students in advance. The evaluation framework also incorporates elements of self-assessment and peer assessment, encouraging students to reflect critically on their own learning and on the contributions of their team members. This reflective dimension is particularly relevant for the development of creative and transversal competences.

Overall, the Design Thinking: Creative Engineering Methods module developed by the Spanish partner represents a comprehensive and practice-oriented approach to fostering creativity in engineering education. By integrating theoretical foundations, creative methodologies, project-based learning and aligned assessment within an e-learning environment, the module demonstrates how design thinking can be effectively embedded in engineering curricula.

The results suggest that this approach may contribute to enhancing students’ creative capacity, collaborative skills and readiness to address complex engineering challenges, although further empirical validation in different institutional contexts would be required.

*3.2.3 Digital thinking: Problem solving (Turkey)*

The module Digital Thinking: Problem Solving, developed by the Turkish partner, addresses the need—identified in the comparative curriculum analysis—to strengthen the explicit development of structured problem-solving competences within engineering education. Although problem solving is a core element of engineering curricula, the analysis revealed that it is often approached from a predominantly technical perspective, with limited integration of creative and reflective dimensions.

In response, this module conceptualises problem solving as a transversal competence that combines analytical reasoning, creativity, ethical awareness, and practical application. Its structure reflects the CEDE competence framework and is designed to guide students from problem identification to solution implementation and reflection through a structured and iterative process.

The first section, “Understanding the Challenge”, focuses on the definition and theoretical foundations of creative engineering problem solving. Students are introduced to the significance of problem-solving methodologies, distinguishing between finding any solution and finding the best solution within specific constraints. Emphasis is placed on the initial phase of problem identification, where students learn to recognize technical limitations, system failures, and emerging societal needs.

Key analytical tools are central to this phase. Students acquire the skills to apply SWOT analysis (Strengths, Weaknesses, Opportunities, Threats) to identify internal and external factors affecting a project. Furthermore, Root Cause Analysis is taught as a critical technique to trace problems to their fundamental sources rather than merely addressing symptoms. Through the analysis of real-world engineering challenges, students develop the competence to define key elements and constraints, ensuring a deep understanding of the context before attempting to solve the problem.

The second section, “Creative Ideation and Solution Development”, shifts the focus to the generation of innovative solutions. This unit is designed to instill the essential skills required to generate groundbreaking ideas and craft effective prototypes. Ideation is framed as a creative process of generating, developing, and communicating new ideas without immediate constraints.

Students are introduced to diverse ideation techniques such as Brainstorming, Mind Mapping, and SCAMPER (Substitute, Combine, Adapt, Modify, Put to another use, Eliminate, Reverse). These methods are practiced within a collaborative environment to encourage divergent thinking. Following ideation, the module emphasizes Prototyping as a pivotal stage in transitioning from theoretical concepts to tangible applications. Students engage in hands-on activities, utilizing materials and tools, ranging from modelling clay to 3D printing and CAD software, to visualize and refine their design concepts. This phase allows for the evaluation of solutions based on criteria such as feasibility, cost-effectiveness, scalability, and innovation.

The third section, “Implementation and Reflection”, is dedicated to the practical execution of engineering projects and the continuous improvement of professional practice. This unit covers the fundamentals of project management, requiring students to create comprehensive project plans, timelines, and resource assessments.

Beyond technical implementation, this section fosters a strong ethical framework. Students are trained to recognize and discuss the ethical implications of their engineering decisions, applying ethical decision-making frameworks to real-world scenarios. The module concludes with a focus on Reflective Practice, encouraging students to critically review their problem-solving journeys, identify areas for improvement, and develop strategies for continuous professional development. Emerging technologies, such as Artificial Intelligence (AI) and Machine Learning (ML), are also explored for their potential to optimize designs and predict outcomes, ensuring students are prepared for future engineering landscapes.

From a methodological perspective, the module adopts an active and student-centred learning approach aligned with CEDE design-based framework. Teaching strategies combine theoretical inputs with problem-based and collaborative activities, enabling students to engage with real-world scenarios and iteratively refine their problem-solving strategies. Collaborative learning is integral, realized through interactive workshops where students work in teams to apply SWOT analysis, brainstorm ideas, and develop prototypes. Simulation-based learning is also utilized, allowing students to engage with realistic project planning scenarios and computer-based problem-solving exercises.

Assessment is aligned with the defined learning outcomes and aims to capture different dimensions of competence development. A combination of continuous assessment and a final theoretical-practical test is used to evaluate students’ ability to apply problem-solving methodologies and justify their decisions. Reflective tools, such as learning journals, are incorporated to support critical thinking and self-assessment.

Overall, this module illustrates how structured problem-solving approaches can be integrated with creativity and reflective practice in engineering education. In line with the findings of the comparative analysis and stakeholder feedback, it offers a coherent framework for addressing complex engineering challenges, although further empirical evaluation would be required to assess its impact on student learning outcomes.

*3.2.4 Digital thinking: Digitalisation and artificial intelligence (Greece)*

The module Digital Thinking: Digitalisation and Artificial Intelligence, developed by the International Hellenic University (IHU) within the CEDE project, addresses the need—identified in the comparative curriculum analysis—to strengthen the integration of digitalisation and artificial intelligence within engineering education. While these topics are increasingly incorporated into curricula, the analysis indicated that they are often addressed in a fragmented and tool-oriented manner, with limited integration of transversal competences such as creativity, critical thinking, and ethical awareness ( Provost & Fawcett, 2013; Sammut & Webb, 2017).

In response to these findings, the IHU module conceptualises digitalisation and AI as socio-technical phenomena that fundamentally reshape industries, organisations, and everyday life, rather than as isolated technological domains. The main objective of the module is to foster digital thinking as a transversal competence that integrates technical understanding, analytical reasoning, creativity, and reflective judgement. This pedagogical orientation is consistent with contemporary perspectives in data science and AI education, which emphasise interpretability, contextual understanding, and responsible application of digital technologies ( Kelleher et al., 2015; Provost & Fawcett, 2013).

The module is designed as a complementary learning unit that can be integrated into existing engineering programmes at an early or intermediate stage of study. It is delivered through the CEDE e-learning platform and structured into six sessions of two hours each (12 hours in total), allowing flexible implementation according to institutional needs.

The learning outcomes of the module are aligned with the CEDE competence framework and focus on the development of digital, analytical, and creative competences relevant to contemporary engineering practice. Upon successful completion of the module, students are expected to:

• Understand fundamental concepts of digitalisation and artificial intelligence and their historical evolution ( Bishop, 2006; Sammut & Webb, 2017).
• Analyse the role of data as a core resource in digital transformation processes ( Han et al., 2011; Provost & Fawcett, 2013).
• Apply basic data analytics and data visualisation techniques to support problem solving and decision making ( Han et al., 2011; Kelleher et al., 2015).
• Distinguish between different types of AI systems and learning algorithms and evaluate their applications across sectors ( Bishop, 2006; Pedregosa et al., 2011).
• Critically assess ethical, societal, and professional implications of AI and digital technologies ( Sammut & Webb, 2017).
• Integrate digital and AI-driven approaches into business and engineering contexts in a creative and responsible manner ( Provost & Fawcett, 2013).

These learning outcomes reflect the module’s emphasis on digital thinking as a competence that extends beyond technical skills to include critical analysis, creativity, and ethical awareness, thereby preparing future engineers for complex, data-driven professional environments.

The module is organised into six interrelated sessions that progressively guide students from conceptual understanding to applied and reflective engagement with digitalisation and artificial intelligence. This structure ensures coherent learning progression by integrating theoretical foundations, practical activities, and critical reflection, supporting the development of digital thinking as a transversal engineering competence.

The first session introduces the core concepts of digitalisation and artificial intelligence, including key definitions, historical developments, and the growing relevance of digital technologies in contemporary society ( Bishop, 2006; Sammut & Webb, 2017). Digitalisation is presented as a transformative process that reshapes economic sectors, organisational models, and professional practices.

The second session focuses on digital thinking and digital transformation. Students explore established frameworks and models for analysing organisational change in digital contexts, with case-based discussions highlighting the importance of critically evaluating technological adoption beyond purely technical considerations ( Provost & Fawcett, 2013).

The third session addresses the central role of data in digital transformation processes. Through hands-on activities on the CEDE e-learning platform, students work with structured and unstructured datasets and engage in data analytics and visualisation tasks using tools such as Orange Data Mining ( Han et al., 2011; Orange Data Mining Team, 2023). These activities strengthen data literacy, model evaluation skills, and evidence-based reasoning ( Kelleher et al., 2015).

Building on this analytical foundation, the fourth session introduces principles and applications of artificial intelligence. Students examine distinctions between machine learning and deep learning and engage in applied case studies comparing supervised learning algorithms using evaluation metrics such as accuracy, precision, recall, and AUC ( Bishop, 2006; Pedregosa et al., 2011). This applied focus supports transparent and responsible interpretation of AI model performance.

The fifth session focuses on the integration of digitalisation and AI within organisational and business contexts. Digital business models, data-driven strategies, and AI-enabled innovation are examined to illustrate creative and strategic applications of digital technologies in professional practice ( Provost & Fawcett, 2013).

The final session adopts a forward-looking and reflective perspective by exploring emerging technologies and future trends alongside ethical, societal, and professional challenges related to digitalisation and artificial intelligence. This session reinforces responsible and critical engagement with digital technologies, consistent with contemporary debates on the societal impact of AI ( Sammut & Webb, 2017).

The module adopts an active, student-centred, and competence-oriented pedagogical approach aligned with the CEDE methodology. Teaching strategies combine short theoretical inputs with case studies, guided discussions, practical exercises, and collaborative activities. Emphasis is placed on linking abstract concepts to real-world engineering and business scenarios, thereby supporting experiential and applied learning ( Kelleher et al., 2015).

The CEDE e-learning platform supports both synchronous and asynchronous learning by providing structured access to learning materials, multimedia resources, practical exercises, and assessment activities. Hands-on activities, particularly in data analytics and AI sessions, engage students with commonly used digital tools such as spreadsheets, data visualisation platforms, and introductory analytics environments ( Han et al., 2011; Orange Data Mining Team, 2023).

Assessment in the module follows a competence-based and learning-outcome-oriented approach aligned with the defined learning outcomes. A knowledge-based test evaluates students’ understanding of key concepts related to digitalisation, data analytics, and artificial intelligence ( Bishop, 2006). A practical assignment or portfolio documents students’ engagement with data analysis, visualisation, or AI-related case studies, enabling assessment of applied competences and creative problem solving ( Pedregosa et al., 2011). Reflective activities, such as short reports or discussion contributions, assess students’ ability to critically evaluate ethical and societal implications of digital technologies ( Sammut & Webb, 2017).

Formative self-assessment tools are embedded throughout the module, including structured questionnaires and reflective journals that support students in evaluating their understanding of digitalisation, data analytics, and AI concepts. Quizzes, scenario analyses, and applied projects promote continuous reflection and competence development, reinforcing digital thinking as a transversal engineering competence.

Overall, the Digital Thinking: Digitalisation and Artificial Intelligence module contributes a critical dimension to the CEDE framework by integrating technical understanding with critical thinking, creativity, and ethical reflection. Initial implementation feedback suggests that digital thinking can be introduced and developed within an e-learning environment and that students value approaches that connect digital technologies with real-world challenges and societal implications ( Provost & Fawcett, 2013; Sammut & Webb, 2017).

*3.2.5 Soft Skills (Slovakia)*

The Soft Skills module, developed by the Slovak partner, focuses on communication as a core transversal competence that underpins creativity, collaboration, and innovation in engineering education. Findings from the comparative curriculum analysis conducted within the CEDE project indicate that, while engineering curricula traditionally emphasise technical knowledge, analytical reasoning, and problem-solving, communication competences are often treated as secondary or implicitly embedded within technical courses.

The comparative analysis conducted within the CEDE project revealed that, in many engineering programmes, soft skills—particularly communication skills—are insufficiently structured, rarely assessed explicitly, and often left to be developed informally through project work. This gap is especially critical in creative engineering contexts, where innovation emerges through interaction, dialogue, negotiation, and shared understanding. In response, the Slovak partner designed a comprehensive Soft Skills module with a strong emphasis on communication for creative engineering, positioning communication not as an auxiliary competence but as a foundational element of creative and innovative engineering practice.

The module conceptualises communication as a multidimensional competence encompassing written, verbal, interpersonal, and digital communication skills, as well as ethical and responsible communication. Its main objective is to equip engineering students with the ability to communicate complex technical ideas effectively, collaborate within diverse teams, and present innovative solutions in a clear, persuasive, and socially responsible manner.

Communication is framed as a dynamic process that enables creativity by facilitating idea generation, critical discussion, feedback, and shared problem-solving. In creative engineering, where solutions often arise from interdisciplinary interaction and iterative design processes, effective communication becomes a key enabler of innovation. The module therefore aims to develop students’ capacity to express ideas clearly, listen actively, adapt messages to different audiences, and engage constructively in collaborative and professional contexts.

The module is structured into three main thematic blocks, each addressing a specific dimension of communication in creative engineering practice. The content is designed to progress from foundational communication skills to advanced applications in professional and innovation-oriented contexts.

The first block introduces communication skills as a cornerstone of creative engineering. Students explore the role of communication in engineering projects, design thinking processes, and innovation ecosystems. Emphasis is placed on understanding communication as more than the transmission of information; it is presented as a relational and interpretative process that builds shared meaning, trust, and collaboration among team members and stakeholders.

A key focus is placed on the ability to translate technical concepts into language accessible to non-technical audiences. Engineers frequently interact with managers, clients, investors, policymakers, and the general public, and their capacity to explain ideas clearly can significantly influence decision-making and project success. Students learn strategies for avoiding excessive technical jargon, using analogies and storytelling, and highlighting the relevance and benefits of engineering solutions rather than focusing solely on technical details.

Active listening is introduced as an essential communication skill in creative engineering. Through guided exercises, students learn how to solicit and incorporate feedback, recognise different perspectives, and engage in constructive dialogue. Active listening is framed as a prerequisite for effective collaboration, conflict prevention, and continuous improvement within engineering teams.

The module also emphasises the use of visual communication techniques—such as sketches, diagrams, prototypes, graphs, and infographics—as powerful tools for conveying complex ideas. Visualisation is presented as a bridge between disciplines, enabling engineers to communicate concepts efficiently and support collaborative decision-making processes.

The second block focuses on effective written and verbal communication in engineering contexts. Written communication is presented as a permanent and strategic component of engineering work, essential for documenting ideas, decisions, and processes. Students are introduced to principles of clarity, precision, structure, and conciseness in technical writing. Particular attention is given to audience-oriented writing, encouraging students to adapt tone, style, and level of detail according to the intended recipients.

The module addresses different forms of written communication relevant to engineering practice, including technical reports, emails, project documentation, and proposals. Students learn how to organise information logically, use headings and visual elements effectively, and ensure accuracy and consistency. Ethical considerations, such as proper attribution of sources and avoidance of misleading statements, are integrated into the discussion of written communication.

Verbal communication is explored as a critical skill for teamwork, leadership, and professional interaction. Students examine elements such as clarity of expression, tone of voice, non-verbal communication, and confidence in delivery. The module highlights the role of verbal communication in mitigating conflicts, enhancing motivation, and fostering innovation within teams. Through practical activities, students develop skills in explaining ideas orally, participating in discussions, and providing constructive feedback.

Public speaking and presentation skills form a key component of this block. Engineers are increasingly required to present ideas in academic, industrial, and entrepreneurial settings. The module introduces principles of effective presentation design, audience engagement, and storytelling. Students learn how to structure presentations logically, use visual aids responsibly, and adapt their delivery to different contexts.

The third block addresses interpersonal communication, collaboration, and ethical responsibility in engineering communication. Students explore how professional relationships are built on trust, respect, transparency, and accountability. The module emphasises that strong interpersonal communication skills are essential for effective teamwork and for creating an environment conducive to creativity and innovation.

Collaboration is presented as a necessity rather than an option in modern engineering practice. Students analyse the dynamics of multidisciplinary teams and learn strategies for effective coordination, role clarification, and shared decision-making. Conflict resolution and negotiation techniques are introduced, highlighting conflicts as natural and potentially productive elements of team interaction when managed constructively. Students practise techniques such as active listening, reframing, and win–win negotiation approaches.

Cross-cultural communication is addressed as a critical competence in global engineering environments. Students examine how cultural differences can influence communication styles, decision-making, and collaboration. The module promotes cultural awareness, empathy, and adaptability as key attributes for engineers working in diverse teams and international contexts.

Ethical and responsible communication is integrated throughout the module. Students are introduced to principles of honesty, accuracy, transparency, and respect in technical communication. Issues related to confidentiality, data protection, and responsible dissemination of information are discussed, particularly in relation to digital communication tools and public presentations. Engineers’ societal responsibility to communicate risks, limitations, and uncertainties clearly is emphasised, reinforcing the link between ethical communication and professional integrity.

From a methodological perspective, the Soft Skills module adopts an experiential, student-centred, and reflective learning approach, in line with the CEDE design-based framework. Teaching strategies combine short theoretical inputs with interactive activities, role-playing scenarios, case studies, group discussions, and guided reflection. This approach aligns with the broader CEDE philosophy of learning by doing and supports the development of communication skills through practice and feedback.

Role-playing exercises are a central pedagogical tool within the module. Students engage in simulated scenarios such as team conflicts, stakeholder meetings, technical explanations for non-expert audiences, and startup pitching sessions. These activities allow students to experiment with different communication strategies, reflect on their effectiveness, and develop confidence in real-world communication situations.

The e-learning platform plays a crucial role in delivering the module. All learning materials, exercises, and assessment tasks are hosted on the platform, enabling flexible access and supporting both synchronous and asynchronous learning. Online discussion forums and collaborative tools facilitate peer interaction and collective learning, while reflective tasks support individual learning trajectories.

Assessment within the Soft Skills module is aligned with the competence-based framework of the CEDE project. Three complementary assessment tools are used to capture different dimensions of communication competence development. A reflective self-assessment questionnaire encourages students to evaluate their communication strengths, learning progress, and areas for improvement. A group-based task assesses interpersonal communication and collaboration skills within team activities. Finally, a presentation-based assessment evaluates students’ ability to articulate ideas clearly, structure arguments, and communicate professionally in oral form.

Assessment criteria are defined through transparent rubrics shared with students in advance. The emphasis is placed on both process and outcomes, reinforcing the understanding of communication skills as competencies that develop over time through practice, reflection, and feedback.

Overall, the Soft Skills: Communication for Creative Engineering module provides a structured approach to integrating communication competences into engineering education. It illustrates how communication can be explicitly addressed as a transversal competence within the CEDE framework.

In line with the findings from the comparative analysis and stakeholder feedback, the module highlights the relevance of communication skills for creative and collaborative engineering practice. While the results indicate its alignment with identified needs, further implementation and evaluation would be required to assess its impact on student learning outcomes.

### 3.3 Results of the round table and stakeholder feedback

The validation of the CEDE training proposal was complemented with a structured stakeholder round table organized in the five countries participants. Here we show the results obtained in the round table organized by the UNIVERSITY OF A CORUÑA. Results are very similar in the different universities of the partners.

The round table took place at the Campus of Ferrol (University of A Coruña). In total, 18 participants attended, representing a deliberately diverse set of perspectives relevant to curricular adoption and employability. The participant profile distribution reported was: 1 academic responsible, 5 teaching staff, 3 companies, 5 student representatives, and 4 “others” (including engineering associations and other associations). In terms of composition and independence, the validation exercise was carried out by a group of professors from UDC who were not involved in the CEDE project, reinforcing the intention to gather external-to-the-project academic feedback. The round table also included students from the two engineering schools on the Ferrol Industrial Campus and representatives from engineering colleges/associations. From the non-academic stakeholder side, companies explicitly reported as participating in the testing process were MINT, Gasóleos San Marcos, Anta Norte, and Tecnopro Hispania.

The discussion “focused on the review and validation of five training modules for incorporation into the curricula of various engineering degrees across Europe”. The round table was therefore framed not as an open-ended ideation workshop, but as a structured validation of (i) the adequacy of theoretical content, (ii) the balance between theory and practice, (iii) the suitability of assessment tools, (iv) scheduling/time allocation, (v) perceived usefulness of the web platform, (vi) coverage of competences, and (vii) feasibility of curricular integration.

To support a shared understanding, the organisers used a dedicated presentation that briefly introduced the CEDE project rationale—i.e., the need to teach and foster creativity in engineering education—and positioned CEDE as a multi-country Erasmus+ cooperation partnership. The slides also contextualised the five-module structure of “Creative engineering” (critical thinking, design thinking, digital thinking/problem solving, digitalisation & AI, and soft skills), explaining that each module was designed with content, methodology and evaluation components. This ensured that stakeholders could comment not only on topic relevance, but also on implementability and assessment design. Stakeholder feedback was captured through a structured questionnaire implemented in Microsoft Forms and also reproduced in the round table presentation The survey included an initial profiling question and seven content/implementation questions:

• Participant collective (Academic responsible/Teaching staff/Company/Engineering associations /Student representatives/Others).
• Whether additional theoretical questions should be added to the modules, and if yes, which ones.
• Whether additional activities should be included (or comments if not).
• Whether the evaluation tools are appropriate, and suggestions for additional ones if needed.
• Whether the schedule/time allocation is appropriate, and comments if not.
• Whether the webpage/platform is useful, including perceived drawbacks.
• Whether additional competences should be added, and which ones.
• Whether it would be possible to include the modules in the academic program, and if not, why.

This combination of (a) respondent profiling and (b) targeted questions on content, pedagogy, assessment and implementation feasibility produced feedback that could be directly mapped onto module redesign decisions and curricular integration strategies.

*3.3.1 Overall results and key insights from stakeholder feedback*

• Sufficiency of theoretical content (Q2)

Across respondents, the prevailing view was that the theoretical content is broadly sufficient. The round table report summarises that “most respondents believe the current theoretical content is sufficient,” with one suggesting a reinforcement via design thinking to better address complex contemporary contexts. This is consistent with qualitative comments in such as: *“I consider the theoretical part is enough, even extensive”* and *“They are in line with what the project proposes.”*

However, one of the most substantive suggestions was to consider “the introduction of design thinking … emphasizing the current reality defined by VULCA-BANI contexts,” connecting module framing to contemporary complexity and uncertainty. Another participant nuanced the need for additional theory by noting that, depending on the cohort, “students already have some kind of background or interest that can help lay the foundation for the module.” Taken together, the feedback supports maintaining the existing theoretical backbone while allowing flexible adaptation depending on prior knowledge and local curricula.

• Need for additional activities and practice-oriented reinforcement (Q3)

A recurring theme was the desire to expand the practical component to strengthen application and skills development. The round table report notes that respondents suggested *“more extensive practical activities or internships”* to apply theoretical knowledge, while also acknowledging time constraints. This is mirrored by comments such as: *“I think the practical part should be a little more extensive … [but] I do not know what extent the students would have enough time to develop the projects during the hours indicated in the schedules”* and *“it would be feasible to implement internships in order to improve the acquisition of the skills.”*

From an implementation standpoint, this feedback points to a trade-off that stakeholders explicitly recognised: increasing hands-on work can improve learning-by-doing outcomes, but it must be reconciled with timetable constraints and realistic student workload.

• Assessment design and integrity considerations (Q4)

While stakeholders generally considered the evaluation tools “appropriate,” they provided several targeted recommendations. The round table report highlights proposals to: incorporate peer assessment, clarify portfolio requirements, use more reflective or reasoning-based questions to prevent misuse of AI tools, and consider alternative credentialing approaches such as open badges. Some recommendations are:

• “ *Yes but we should include peer to peer assessment.*”
• A detailed critique notes that the “portfolio” concept may be confusing (a portfolio is usually built over time), and that “solving a challenge is the best way to internalize and put into practice” design thinking; the comment also proposes “the inclusion of an open badge system … encouraging students to self-assess,” referencing experience from another Erasmus+ project (“Open Design and Manufacturing”).
• Multiple participants raised concerns about online multiple-choice questionnaires being vulnerable to generative AI: one warns that students could “ *pass all the questions to an artificial intelligence chat*,” recommending questions that require a “high degree of student reasoning” or essay-based approaches when theoretical knowledge is assessed.

These comments are especially relevant for e-learning deployment: they do not reject quizzes outright, but they call for assessment redesign that protects academic integrity and better evaluates reasoning, reflection and authentic creative problem solving.

• Schedule and balance of emphases (Q5)

Stakeholders generally found the schedule appropriate, but feedback emphasised the importance of balancing breadth and depth and adapting timing to different cohorts. The round table report states that respondents “generally find the schedule appropriate,” though some suggested adjustments to balance content depth, emphasise creativity over communication, and tailor timing based on prior knowledge and engineering focus.

The qualitative feedback provides texture to this: *“It looks like a tight schedule to cover a big topic, but the approach looks promising,”* and *“it might be necessary to place greater emphasis on the first part … or the second … depending on the type of engineering and prior knowledge.”* One of the most pointed comments argued that *“communication skills have a great weight … Creativity and knowledge shall prevail over communication,”* warning that *“a wrong response well communicated can be dangerous, because [it can] transmit false trust to [the] general public.”* This comment is important because it reframes “soft skills” not as an end in themselves, but as something that must be anchored in correctness, responsibility, and critical judgement—particularly relevant in technology-mediated contexts.

• Web platform usefulness (Q6)

For the question on the webpage/platform, results notes that *“It was no possible to answer this question.”* As this is the only explicit outcome recorded for Q6 in the provided materials, conclusions about perceived platform usefulness must remain cautious: the available dataset does not contain evaluative responses, only the indication that it could not be answered.

• Competences to be added (Q7)

While one respondent considered the competences sufficient, others suggested reinforcing specific transversal and cognitive skills. The round table report identifies additions such as complex problem solving, empathy, testing and prototyping, and the ability to critically assess incorrect or AI-generated responses. This is consistent with direct comments: *“Ability to quickly check and judge incorrect solutions … detect AI-related bad responses,”* and *“The DT module could include … Complex Problem Solving … listening and empathy … focus on testing and prototyping for solution validation.”*

These suggestions are coherent with the project’s overarching aim to strengthen creative engineering profiles: stakeholders are effectively asking for competence framing that makes explicit the link between creativity, critical judgement, and validation practices (prototype/test), as well as human-centred skills (listening/empathy).

• Feasibility of curricular integration (Q8)

Finally, stakeholders were broadly optimistic about integrating the modules into academic programmes, but emphasised that the most realistic path is often flexible or optional integration rather than a one-size-fits-all compulsory subject. The round table report summarises that most respondents believe inclusion is possible, *“often as optional, complementary, or adaptable components,”* while noting challenges due to curriculum constraints and the need to develop transversal skills.

Qualitative statements reinforce this position: *“Yes … it would help students of more ‘technical’ engineering programs develop new skills,”* but also *“No … better if it’s included in a non-compulsory activity program,”* and *“Yes, like an optional subject.”* Several participants propose modular integration strategies: integrating single modules “as optional training courses that complement the academic program,” or using the content as “basic training” that can serve as a foundation for later development across the programme, even while acknowledging implementation complexity. One respondent also suggested strengthening collaboration with companies or analysing success stories (“Collaborative module with companies or analysis of success stories”).

*3.3.2 Synthesis*

Taken as a whole, the round table and survey responses validate the relevance of the CEDE modules while identifying concrete, actionable refinements (summarised in Table 1). First, the theoretical base is largely endorsed, with a targeted call to ensure the framing is robust for contemporary complexity (e.g., links to design thinking and complex problem contexts). Second, stakeholders consistently request stronger practice-oriented learning opportunities (expanded practical tasks and/or internships), but with awareness of schedule feasibility. Third, assessment design emerges as a critical area for improvement: respondents support adding peer assessment, clarifying portfolio/journal expectations, and redesigning quiz-based assessment to require reasoning and reduce vulnerability to generative AI misuse; open badges are proposed as a motivating complementary mechanism. Fourth, the schedule is seen as workable but may need adaptation by degree type and prior knowledge, and stakeholders highlight the need to preserve a clear hierarchy where creativity and correctness underpin communication. Finally, most stakeholders see curricular integration as feasible, particularly through flexible models such as optional subjects, complementary modules, or embedding selected units within existing courses—an approach aligned with the diversity of European engineering curricula.

**Table 1.** Key insights derived from the CEDE stakeholder round table.

Dimension evaluated	Main trend	Illustrative comments
Theoretical content	Largely sufficient	“The theoretical part is enough, even extensive”
Practical activities	Need for expansion	“The practical part should be more extensive”
Assessment tools	Appropiate, but improvements suggested	“Include peer assessment”; “Avoid AI-vulnerable quizzes”
Schedule	Generally appropiate	“Tight but promising”
Competences	Some additions suggested	“Complex problem solving”; “Empathy”
Curricular integration	Feasible with flexibility	“Optional or complementary modules”

## 4. Conclusions/discussion

The CEDE – Creative Engineering project provides a structured and collaborative approach to integrating creativity-related competences into engineering education through a coordinated, international and modular framework. The results of the project indicate the relevance and potential feasibility of such an approach, based on comparative curriculum analysis, instructional design, and participatory evaluation.

By incorporating design thinking, creative engineering methods, critical thinking, and digital thinking, the project proposes an approach aligned with the growing demand for engineers capable of addressing complex, real-world problems. The results suggest that fostering creativity through structured methodologies may support the development of both technical and transversal competences.

### 4.1 Key findings

The primary outcome of the CEDE project is the development of a common complementary module designed to foster creative engineering competences across diverse higher education contexts. The module is composed of five interconnected thematic areas—critical thinking, design thinking, digital problem solving, digitalisation and artificial intelligence, and soft skills—each addressing a specific dimension of creativity in engineering practice. Together, these modules form a coherent framework that supports the development of creativity as a transversal competence embedded within engineering education rather than as an isolated or optional component.

The comparative analysis of engineering curricula conducted at the initial stage of the project highlighted that while creativity is often mentioned in educational objectives, it is rarely explicitly embedded into the core curriculum. Also, it revealed significant variability in how creativity and transversal competences are addressed across institutions and countries. In many cases, creativity was present only implicitly or fragmented across courses, with limited use of active and student-centred methodologies. These findings confirmed the need for an explicit, structured and transferable educational intervention that prioritise creativity as a fundamental skill in engineering education.

The CEDE framework responds to this need by offering a flexible module that can be integrated into existing curricula at an early stage and adapted to different institutional and disciplinary contexts. Furthermore, the roundtable feedback from students, faculty, and industry stakeholders indicates that creativity-focused modules were perceived as highly beneficial for improving students’ problem-solving abilities, teamwork skills, and capacity for innovation.

### 4.2 Discussion: Implications for engineering education

The integration of creativity and design thinking into engineering curricula represents a paradigm shift in the way we view the role of engineers. Traditionally, engineering education has focused heavily on technical knowledge and analytical skills, with creativity often regarded as a supplementary or non-essential component. However, as the demand for engineers capable of addressing complex, ill-defined problems continue to grow, it is becoming increasingly clear that creativity is no longer a luxury but a necessity.

From a pedagogical perspective, the results of the CEDE project highlight the importance of active, project-based and student-centred learning methodologies for the development of creative competences in engineering students. One of the main challenges identified throughout the project was the need for pedagogical approaches that balance creativity with the rigorous technical requirements of engineering education. The CEDE project suggests that creativity and technical skills can be integrated within engineering education, supporting more holistic approaches to problem solving. However, the process of integrating creative methodologies into traditional engineering courses is not without challenges. It requires both faculty and students to embrace new ways of thinking, often disrupting established teaching methods. Moreover, ensuring that creativity is evaluated in a meaningful and measurable way remains a critical challenge for educators.

Across all modules, creativity is approached as a competence that can be taught, practised and assessed through structured methodologies rather than as an innate or informal ability. The project provides preliminary evidence that creativity can be addressed and assessed through structured methodologies, that integrate critical thinking, design thinking, digital tools and soft skills.

The use of a shared e-learning platform emerges as a key enabler of this approach. The platform is designed to facilitate access to learning materials, support collaboration and reflection, and enable the integration of diverse assessment tools. It also provides a structure that support coherence across modules while allowing adaptation to different contexts. This balance between standardisation and flexibility is one of the strengths of the CEDE framework.

The development of appropriate assessment tools is essential for ensuring that students’ creative competences are effectively monitored and evaluated. The development of fifteen assessment tools, aligned with learning outcomes and creative competences, responds to a recognised challenge in engineering education: the meaningful evaluation of creativity and transversal skills. By combining formative and summative approaches, it offers a structured framework for assessing both processes and outcomes.

Furthermore, the CEDE project highlighted the importance of cross-institutional and international collaboration in educational innovation. The engagement of five partner institutions across Europe allowed for a rich exchange of ideas, pedagogical strategies, and cultural perspectives on creativity in engineering education. This collaborative approach not only enriched the content and methodology of the creative engineering modules but also facilitated the development of an e-learning platform that can be easily adapted to different institutional contexts and engineering disciplines.

### 4.3 Dissemination of the Project

The project’s dissemination strategy combined institutional communication, mass-media outreach, and community-facing events to ensure visibility at local, national, and EU levels across partner countries. Activities were coordinated through university channels, public events, and the project’s own EU-wide social media presence, with documentation (posts, news items, videos) openly accessible.

UNIMOL implemented a dual track of public events and digital communication. A national, online multiplier event engaged stakeholders in creative engineering (>40 participants). Multiple Open Day activities in Campobasso showcased project outcomes to university audiences (>100–200 attendees). A Research Day in Isernia (>200 participants) further extended reach among students and staff. These were amplified through social media posts at institutional and project pages ensuring EU-level discovery and sustained visibility.

Spain prioritized institutional web and regional/national media to reach wider publics beyond academia. UDC’s newsroom published multiple items summarizing project goals and meetings to a potential audience of >20,000 The project was continuously featured on campus social media with engagement metrics. Press coverage spanned local and national outlets The project also reached radio audiences. Finally, the project was presented in a national teaching innovation conference (XIGAID).

Dissemination in Slovakia combined large-scale fair presence and STEM engagement with students. The Agrokomplex 2024 fair in Nitra reached a general audience in the tens of thousands (>10,000). Additional STEM hackathon and workshops targeted the educational community (≈30–35 participants per activity). These actions were complemented by social media posts—in Slovak and English—announcing project meetings to national/EU audiences (e.g., Facebook items with bilingual coverage and links).

Greek dissemination activities focused on enhancing the visibility of project meetings and strengthening regional outreach through institutional and partner communication channels. Project meetings held in Serres were highlighted via campus communication platforms and were subsequently referenced in press notes issued by Spanish partners, illustrating effective cross-border information flows and the generation of lasting digital records of the project activities.

In Turkey, Kocatürk Consulting contributed concretely to the project’s dissemination by actively participating in project meetings and providing content support for the promotion of project outputs. In particular, the partner shared information highlighting developments related to the e-learning platform through the consortium’s communication channels and organised a national multiplier event targeting key stakeholders (academics, engineering educators, university staff, students, and industry/SME representatives). Through this event, Kocatürk Consulting promoted the CEDE outputs and supported the practical use of the e-learning platform and creativity-focused modules, thereby fostering the uptake of the project results in Turkey and their integration into educational practices beyond the project period.

An English-language Facebook page functioned as a cross-partner hub for videos, meeting summaries, and poster highlights, extending discoverability across the Erasmus+ ecosystem. Collectively, these activities demonstrate a balanced portfolio: mass-event exposure (Italy, Slovakia), sustained digital amplification (Italy, Spain, EU page), institutional credibility via university newsrooms (Spain), and media corroboration (press and radio), meeting the typical Erasmus+ expectations of multi-level dissemination.

### 4.4 Lessons learned and future directions

Several key lessons emerged from the CEDE project, which may guide future efforts in integrating creativity into engineering education:

First, integrating creativity-focused modules into engineering curricula requires institutional commitment and coordination among teaching staff. While the modular and complementary nature of the CEDE framework facilitates integration, long-term sustainability depends on the willingness of institutions to embed such approaches within their educational strategies.

The success of creativity modules in different institutional contexts underscores the importance of adaptability. A one-size-fits-all approach is unlikely to work in diverse educational environments. The CEDE project’s flexible, modular design allows for adjustments based on local curricula, institutional resources, and cultural contexts, ensuring that creativity-focused learning can be integrated effectively into a wide range of engineering programmes.

Second, the successful implementation of creative and student-centred methodologies highlights the need for continuous professional development for educators. Teaching creativity in engineering requires not only new tools and resources but also a shift in pedagogical mindset, moving from content transmission towards facilitation, guidance and reflection.

Third, the participatory evaluation process, particularly the round tables involving students, teachers, university staff, professionals and companies, was identified as a key element for ensuring the relevance and applicability of the developed modules, and it is critical for refining educational content and methodologies. The iterative nature of design thinking aligns well with the need for continuous feedback in education, and this multi-stakeholder engagement strengthened the quality of the outcomes and reinforced the connection between higher education and professional practice.

Feedback from industry professionals has been instrumental in shaping the modules and ensuring their relevance to the skills needed in the workplace. Moving forward, stronger collaboration between universities and industry stakeholders is essential for developing curricula that address both academic and practical needs, fostering innovation and employability.

Looking forward, future work should focus on the long-term evaluation of the impact of the CEDE framework on student learning, employability and professional performance. The development of the e-learning platform has proven to be an effective tool for delivering the module content, fostering collaboration, and assessing student progress. However, further investment in e-learning technologies and resources is needed to expand the reach of such initiatives and ensure that all students, regardless of geographical location or institutional resources, have access to creative engineering education.

Expanding the use of the e-learning platform, integrating the modules into additional engineering programmes and strengthening collaboration with industry partners represent key directions for further development. In addition, future research could explore the scalability of the framework and its adaptation to other disciplines beyond engineering.

## 5. Conclusions

In conclusion, the CEDE project represents a structured and collaborative attempt to integrate creativity and innovative problem-solving methods into engineering education. This project contributes to the discussions by providing a transferable and evidence-informed framework for embedding creativity and transversal competences into engineering curricula.

The findings suggest that structured methodologies such as design thinking and project-based learning may support the development of competences relevant for addressing complex engineering challenges. While the results indicate the relevance and perceived value of this approach, further implementation and longitudinal evaluation would be required to assess its impact on student learning and professional outcomes.

Continued development, dissemination and institutional support will be essential to ensure the sustainability of such initiatives. The lessons learned from the CEDE project will inform future educational practices and contribute to the evolution of engineering education towards more integrated, creative, and student-centred approaches.

## Ethics and consent

In accordance with Spanish Law 14/2007 of 3 July on Biomedical Research, which limits its scope to biomedical research involving human participants or human biological material, as established in Articles 1 and 12 ( https://www.boe.es/eli/es/l/2007/07/03/14/con), ethical approval is not required for this project. In addition, the Ethics Committee of the University of A Coruña indicated that projects not involving interventions on human beings or the use of biological samples of human origin are exempt from the requirement of formal ethical approval. As the CEDE project does not include such interventions, ethical approval was not required.

Nevertheless, all activities were conducted in full compliance with current regulations on personal data protection and information preservation. The handling of information was fully compliant with Organic Law 3/2018 of 5 December on the Protection of Personal Data and the Guarantee of Digital Rights, as well as with Law 37/2007 of 16 November on the reuse of public sector information, which regulates the reuse of research data generated by public institutions, provided that requirements relating to personal data protection, confidentiality, and intellectual property are respected.

( https://www.boe.es/eli/es/l/2007/11/16/37; https://www.boe.es/eli/es/lo/2018/12/05/3).

Furthermore, our approach is aligned with the European Commission Recommendation of 17 July 2012 on access to and preservation of scientific information (2012/417/EU), as revised and replaced by Commission Recommendation (EU) 2018/790, which promotes open access to and reuse of publicly funded research results within existing legal and ethical frameworks.

( https://www.boe.es/buscar/doc.php?id=DOUE-L-2012-81299, https://eur-lex.europa.eu/legal-content/ES/TXT/PDF/?uri=CELEX:32018H0790, https://eur-lex.europa.eu/eli/reco/2018/790/oj).

All participants in the round table were adults and were informed at the beginning of the session about the purpose of the study and the applicable data protection legislation. Informed consent was obtained from all participants involved in the activities described in the manuscript. Given the non-interventional and non-biomedical nature of the study, informed consent could have been obtained in a non-written (verbal) form, in accordance with standard practice for this type of research and in the absence of any legal requirement for written consent in this context. Nevertheless, written informed consent was in fact obtained from all participants: written consent forms authorizing the taking and use of photographs were signed; although photographs are not included in this article, consent was explicitly granted for their use for research and publication purposes.

Following the round table, participants completed a questionnaire. The qualitative data presented in this article were derived from anonymized responses, making it impossible to identify individual participants.

## Data Availability

The data supporting the findings of this study are originated from an anonymised stakeholder survey conducted during the CEDE project round table. All relevant aggregated data and qualitative feedback are presented within the article. Due to the nature of the data and in order to preserve participant’s confidentiality, individual-level raw data are not publicly available. Due to ethical restrictions and participant confidentiality, individual-level data from this study cannot be made publicly available. Researchers wishing to access the data may submit a reasoned request to the corresponding author ( cristina.prado.acebo@udc.es), providing a brief description of the intended use of the data. Access will be granted subject to approval by the institutional ethics committee and in accordance with applicable data protection legislation. The training modules developed within the CEDE project and discussed in this study are publicly accessible through the project’s e-learning platform at: http://cede-project.eu/. All data supporting the results of this study are provided in aggregated and anonymised form within the article. Zenodo: Round table results- CEDE project. https://doi.org/10.5281/zenodo.19354330 ( Prado-Acebo et al., 2026b). The project contains the following underlying data:
• CEDE PROJECT SURVEY results.xlsx (Data Set) Data are available under the terms of the Creative Commons Attribution 4.0 International license (CC-BY 4.0). The Creative Engineering (CEDE) project e-learning platform, which has been developed by the authors of this article and hosts the training modules described herein, is publicly available at: http://cede-project.eu/. The platform provides open access to the course structure and learning materials developed within the project. Zenodo: Survey questions for the CEDE project round table. https://doi.org/10.5281/zenodo.19354517 Prado-Acebo et al., 2026a). The project contains the following underlying data:
• CEDE PROJECT SURVEY.pdf (Survey Questions) Data are available under the terms of the Creative Commons Attribution 4.0 International license (CC-BY 4.0). No software was developed or used in this study.
