# Supplementary material for: Fostering creativity in engineering education: The CEDE project and the design of a European e-learning platform
Source: Open Res Eur. 2026 Aug 8;6:159. Originally published 2026 May 11. [Version 3] doi: 10.12688/openreseurope.23240.3 (PMC13650312; doi:10.12688/openreseurope.23240.3)
Supplement: Peer review history

# Reviewer report: Reviewer response for version 3

**DOI:** 10.21956/openreseurope.26779.r78964

**Published:** 2026-10-12

**Authors:** Sharon Guaman-Quintanilla

**Affiliations:** 1 Escuela Superior Politecnica del Litoral, ESPOL, i3lab Entrepreneurship and Innovation Center, Guayaqui, Ecuador

**Overall assessment**

 The topic is timely and relevant. Embedding creativity explicitly in engineering curricula, rather than leaving it as an implicit by-product of technical training, is a recognised need. The transnational, multi-stakeholder nature of the project is a genuine strength. The authors have responded seriously to the previous rounds of review: the research questions, DBR figure, instrument description, cross-partner synthesis and limitations section are clear improvements. The more cautious framing of learning effectiveness in Section 4.5 is also welcome.

 I agree with the points raised by the previous reviewers, particularly Albers & Bastian on the definition of creativity, the missing curriculum analysis template, and the module descriptions. I do not repeat them here. My comments focus on four issues that, in my view, still limit the scientific soundness of the article:

- the extent to which the study actually fulfils a DBR design;
- the conceptual link between the five modules and creativity;
- how design thinking and creativity are facilitated and assessed in an online format;
- internal consistency between methods, results and conclusions.

**1. The DBR claim needs to be substantiated with evidence of iteration and implementation.**

 DBR is characterised by iterative cycles of design, implementation in authentic learning settings, analysis and redesign. It produces two outputs: a refined intervention and transferable design principles (Anderson & Shattuck, 2012; McKenney & Reeves, 2019; Bakker, 2018). Figure 1 depicts feedback loops from Cycles 4 and 5 back to Cycles 2 and 3, but the manuscript does not document any iteration that actually took place. Table 5 mentions “minor refinements introduced before final implementation”, but readers cannot see what was changed.

 I suggest the authors to:

 (a) provide a traceable change log (e.g., a table) linking each stakeholder theme to the concrete design decision taken and the module element that was revised.

 (b) state explicitly whether the modules have been implemented with students in any partner institution. Several statements imply implementation:

- “initial implementation feedback suggests…” (3.2.4);
- the platform “has proven to be an effective tool for delivering the module content, fostering collaboration, and assessing student progress” (4.4).
- If implementation took place, its data should be reported. If not, these statements should be removed, and the study should be described as the analysis and design phases of a DBR process with expert and stakeholder review, rather than a completed DBR cycle;

Please also check the methodological citation in Section 2.1. Laursen & Haase (2019) is cited as the reference for the “qualitative and design-based research (DBR) approach”. That article compares design thinking with designerly thinking; it does not seem as a DBR methodology source.

**2. RQ1 is not answered with data.**

 Section 3.1 is the only section that addresses RQ1, and it reports no findings from the curriculum analysis. It gives no number of programmes analysed per institution, no matrix of explicit and implicit presence of creativity-related learning outcomes, courses or assessment, and no examples. Its claims about global trends and about helping “prospective students choose the program” fall outside the scope of the study.

 Yet the key conclusion, that creativity is “addressed only implicitly or fragmented”, rests entirely on this analysis. Please provide:

- a summary table per institution or country with the criteria of the common template (explicit learning outcomes, courses, teaching methods, assessment);
- the comparative report itself as underlying or extended data.

Please also clarify which Turkish curricula were analysed. The Turkish partner seems like a consulting firm, and Section 2.1 states that the project “involved five higher education institutions”, which contradicts the Introduction’s description of an external partner organisation.

**3. The conceptual model linking the five modules to creativity is missing.**

 The framework is presented as “creativity-oriented”, but the manuscript does not explain how critical thinking, digitalisation and AI, or communication contribute to creativity. Nor does it say which dimension of creativity each module targets. Possible dimensions are person, process, product and press (Rhodes, 1961), or divergent and convergent thinking, problem finding and idea evaluation (Cropley, 2015; Daly, Mosyjowski & Seifert, 2014).

 The previous reviewers asked for a definition of creativity; I would go further and ask for a constructive alignment matrix (Biggs). It would cross each module’s learning outcomes, the creativity dimension addressed, the teaching activities and the assessment tool. Such a matrix would make the framework’s coherence visible; expose overlaps, which are currently substantial. Ideation techniques such as brainstorming and SCAMPER, prototyping, and the DT process itself appear in Modules 1, 2 and 3; Module 1 contains a full “Design Thinking Process” sub-unit that duplicates Module 2; show where creativity is genuinely developed, e.g., in Module 4.

**4. Facilitation of design thinking and creativity in an e-learning format is not addressed.**

 Design thinking is typically learned through team-based, coached, hands-on work on real problems with real users. The quality of facilitation is a critical and under-researched factor. The manuscript describes the platform’s content but not how teaching happens. Please clarify:

- whether the modules are self-paced, blended or teacher-led;
- who facilitates, and with what preparation. The Slovak round table and Section 4.4 themselves point to teacher support as a key need;
- how teams are formed and how online teamwork is supported;
- whether students work with real users or problem owners;
- how prototyping and testing are carried out online.

If a facilitator guide exists, it should be described and shared. This is central to the claims of transferability and of curricular integration.

**5. Assessment of creativity: description and consistency.**

 The article states that fifteen assessment tools were developed, three per module. However, the descriptions in Section 3.2 do not match Table 4. Please reconcile these. More importantly, explain how creativity itself is assessed, beyond knowledge tests. Established options exist, such as the AAC&U VALUE Creative Thinking rubric (Rhodes, 2010), the Consensual Assessment Technique (Amabile, 1982), or multi-source assessment combining self, peer and teacher ratings. Please justify why new tools were preferred over these, as Albers & Bastian also asked, and provide the rubrics as extended data. Since stakeholders flagged that online multiple-choice tests are vulnerable to generative AI, please also state whether and how this was addressed.

**6. Stakeholder data should be reported with frequencies, not only qualitative descriptors.**

 Expressions such as “most participants”, “a large majority” or “generally appropriate” should be replaced or complemented by counts and percentages per item, per country and, where possible, per stakeholder group. This requires only descriptive statistics, but it is necessary for readers to judge the strength of the stakeholder validation.

 To be clear, I am not asking the authors to reframe the study as a mixed-methods design; the qualitative DBR framing is appropriate for its aims. Reporting descriptive frequencies of the closed items would simply make the existing evidence transparent and allow readers to judge the strength of stakeholder agreement. 

**7. Inconsistency regarding the platform evaluation.**

 Section 2.6 states that participants were “invited to explore the platform”. However, at UDC, the most detailed case, the platform question (Q6) “was not possible to answer”. Please explain why. Was the platform not yet available, or was it not accessible during the session? The answer bears directly on how much the platform has been validated, and the conclusions about the platform should be adjusted accordingly.

**8. Qualitative analysis procedure.**

 The analysis is described as inductive thematic analysis. However, the initial codes listed (content adequacy, activities, assessment, platform, competences, curricular integration) map one-to-one onto the questionnaire items, which suggests a deductive or framework analysis. Please clarify this and specify:

- who coded the data, and how many coders were involved;
- whether a codebook was used;
- how disagreements were resolved;
- how responses in different languages were translated and handled across partners;
- how the cross-partner “documentary synthesis” was conducted when the sources were reports rather than raw data.

**9. Conclusions still overstate the evidence.**

 Section 4.5 correctly acknowledges that no learning outcomes were measured, but several statements elsewhere remain stronger than the data allow:

- “proposing and empirically validating the CEDE framework” (Abstract);
- “the findings demonstrate that creativity… can be systematically taught and assessed” (Conclusions);
- creativity-focused modules “were perceived as highly beneficial for improving students’ problem-solving abilities, teamwork skills, and capacity for innovation” (4.1);
- the platform “has proven to be an effective tool” (4.4);
- “the project shows that creativity can be taught in a structured way and that doing so helps to prepare engineers” (Plain Language Summary).

The evidence supports stakeholder-perceived relevance and feasibility of a proposed framework, not validated effectiveness. Please align these statements with Section 4.5.

**10.** **Availability of the e-learning platform and module materials.** 

 The manuscript states that the training modules are “publicly accessible through the project’s e-learning platform at http://cede-project.eu/”, and this URL is listed as the main source of extended data. However, when I attempted to access it (11 October 2026, from different browsers), the domain could not be resolved (DNS error “NXDOMAIN”), so I was unable to examine the platform, the module materials or the assessment tools described in the article. This may be a temporary issue, but since the platform and modules are core outputs of the study, and the project ended in 2025, I recommend that the authors (a) verify and restore access to the platform, and (b) deposit the module materials, assessment rubrics, curriculum analysis template and comparative report in a persistent repository with a DOI (e.g., Zenodo, as already done for the round-table data), ideally under an open licence. Persistent deposit would also address the long-term sustainability of the project outputs discussed in Section 4.4, independently of the maintenance of the project website.

**Minor points**

- Table 2 is titled “Correlation between questionnaire items and the dimensions analysed”; “correspondence” or “mapping” would be more accurate. The text also mentions six dimensions while the table lists seven.
- The workload of each module (hours or ECTS) is reported only for Module 4 (12 hours). Since stakeholders evaluated the schedule (Q5), the time allocation of all modules should be reported.
- The ethics statement relies on Spanish legislation and the UDC Ethics Committee. Please clarify the basis for data collection in the other four countries.
- Some recent work on DT facilitation and on teaching creativity in engineering could strengthen the positioning.

**Final thoughts**

 The CEDE framework is a valuable and practically useful outcome, and the article can make a sound contribution if it is positioned accurately. It is a well-documented account of the analysis, design and stakeholder-review phases of a DBR process. It should be supported by transparent curriculum analysis data, descriptive stakeholder statistics, an explicit link between the modules and creativity, and a traceable record of design iterations. Points 1, 2, 5, 6 and 9 are, in my view, essential. The others would substantially strengthen the manuscript.

Is the study design appropriate and does the work have academic merit?

Partly

Is the work clearly and accurately presented and does it cite the current literature?

Partly

If applicable, is the statistical analysis and its interpretation appropriate?

Not applicable

Are all the source data underlying the results available to ensure full reproducibility?

Partly

Are the conclusions drawn adequately supported by the results?

Partly

Are sufficient details of methods and analysis provided to allow replication by others?

Partly

Reviewer Expertise:

Design thinking in higher education; design thinking facilitation and instructional strategies; assessment of creativity, problem solving and teamwork from a multi-actor perspective; entrepreneurship education; faculty development for non-design facilitators.

I confirm that I have read this submission and believe that I have an appropriate level of expertise to confirm that it is of an acceptable scientific standard, however I have significant reservations, as outlined above.

# Reviewer report: Reviewer response for version 3

**DOI:** 10.21956/openreseurope.26779.r79003

**Published:** 2026-10-08

**Authors:** Albert Albers, Annika Bastian

**Affiliations:** 1 Karlsruhe Institute of Technology, Karlsruhe, Baden-Württemberg, Germany

1) In Introduction paragraphs 5 and 6, sources for relevant information are missing.

 2) The structure of the Introduction does not follow a clear line of argumentation that leads to your project and its results. The 4th paragraph repeats a lot of what has been said, and the entire introduction does not include an understanding of what you actually mean by creativity. It is a term with a variety of different definitions, and to introduce your work you should not start by why it is needed but first by what it is, or means for your project. This might be information that you already stated for your grant agreement, but it is crucial for the readers of this results contribution here.

 3) Placement of Figures. Figure 1 appears in the middle of a sentence. Please place each figure in an appropriate place after its introduction.

 4) Content of Figure 1. Parts of the content of Figure 1 are repeated in the same way in the text. Please remove. Refer to the figure in the appropriate place in the text and let the Figure speak for itself. To do so, please give the columns a headline, e.g., Phases and Results.

 5) The template mentioned in 2.2 is relevant for this contribution and should be included in the annex, since it is the data collection instrument to answer RQ1.

 6) Please show the process of how the data was collected, where it was stored, and how it was synthesized in a figure: 1) collection via analysis template 2) introduction of data in shared data set (include platform or database) 3) analysis of recurring patterns and cross-case comparison (here we need the how) 4) synthesis into comparative report

 7) Why was each module developed by a different partner?

 8) Section 2.4 includes elements that are part of the results, not the methodology

 9) Section 2.5 is missing details of how the assessment takes place and why new tools are developed and not existing tools integrated

 10) 2.6.1 please define what makes the experts experts

 11) 2.6.1 please include Zenodo DOI

 12) Is the dissemination part of a methodology section of a research paper? This is very atypical

 13) “Universities worldwide are beginning to integrate topics like artificial intelligence, renewable energy systems, and data analytics, reflecting industry demands and global challenges.” You are analyzing only the differences between the project partners; please ensure that you stay scientifically precise and do not overstate. If you want to keep this statement in the results section, you need to add a literature source here that states this clearly for more than the participating countries, and then include a literature review also in the methodology section. Alternatively, tone it down and make it sound like what it is: the trend you see among the partners.

 14) Section 3.2 contains the long lines typical of AI —

 15) 3.2.1 is completely missing sources. Even though this is your course program, the statements you make have sources. E.g., creativity is based on Stein 1953 and so on. You can’t state all of this in a scientific paper without giving the relevant sources.

 16) SCAMPER is in module 1 and module 3 – looks like these two models were not so well coordinated

 17) 3.2.4 It is unclear how students actually learn to be creative, apply creative thinking, or anything creativity-related in the Digitalisation module. Creativity is mentioned a few times, but how it is integrated and what students learn concerning creativity remains unclear.

 18) 3.2.5 Please detail some of the strategies for avoiding technical jargon. Concepts in this section are introduced on an abstract level that makes it hard for the reader to understand what the students are actually taught. It is clear what they are supposed to learn, but not how. Which concepts are used, by whom, and why.

 19) In 3.2.5, please use some graphical representation (e.g., for the different blocks) to break up the massive text block

 20) 3.3.1 contains elements that need to be placed in the methodology section

 21) 3.3.1.1 inconsistent display of citations: some cursive, others not

 22) Placement of Table 6 is strange

 23) Table 6 and its surrounding text are repetitive rather than complementary

 24) The differences in data collection formats and available documentation are a limitation that should be discussed in more detail

 25) The dissemination part does not seem relevant to the research questions

 26) 4.4, 4.5, and 5 are repetitive; please compress

Is the study design appropriate and does the work have academic merit?

Partly

Is the work clearly and accurately presented and does it cite the current literature?

Partly

If applicable, is the statistical analysis and its interpretation appropriate?

Not applicable

Are all the source data underlying the results available to ensure full reproducibility?

Yes

Are the conclusions drawn adequately supported by the results?

Partly

Are sufficient details of methods and analysis provided to allow replication by others?

Partly

Reviewer Expertise:

Cultural influences on creativity in product engineering, creativity methods and method adaptation for distributed settings, distributed product engineering, engineering tools, methods and processes

We confirm that we have read this submission and believe that we have an appropriate level of expertise to confirm that it is of an acceptable scientific standard, however we have significant reservations, as outlined above.

# Reviewer report: Reviewer response for version 3

**DOI:** 10.21956/openreseurope.26779.r78798

**Published:** 2026-08-26

**Authors:** Sahin Idil

**Affiliations:** 1 The Scientific and Technological Research Council of Türkiye (TÜBİTAK), Ankara, Turkey

It is seen that the authors have made required revisions.

Is the study design appropriate and does the work have academic merit?

Yes

Is the work clearly and accurately presented and does it cite the current literature?

Yes

If applicable, is the statistical analysis and its interpretation appropriate?

Not applicable

Are all the source data underlying the results available to ensure full reproducibility?

Partly

Are the conclusions drawn adequately supported by the results?

Partly

Are sufficient details of methods and analysis provided to allow replication by others?

Partly

Reviewer Expertise:

My areas of expertise include STEM education, digital education, artificial intelligence in education, curriculum development, design thinking, project-based learning, and the development of transversal competences such as creativity, critical thinking, problem solving, collaboration, and communication. I am able to assess the article particularly in terms of its educational design, methodological clarity, curriculum relevance, e-learning approach, and contribution to engineering education.

I confirm that I have read this submission and believe that I have an appropriate level of expertise to confirm that it is of an acceptable scientific standard.

# Reviewer report: Reviewer response for version 2

**DOI:** 10.21956/openreseurope.26362.r76616

**Published:** 2026-07-09

**Authors:** Ida Nugroho Saputro

**Affiliations:** 1 Sebelas Maret University, Surakarta, Central Java, Indonesia

Abstract

 1.The abstract focuses more on describing the project activities than presenting the scientific findings of the study. Readers are not provided with clear information regarding what was measured, how the data were analyzed, and what the main findings are.

 2.The novelty of the study is not explicitly stated. The authors should add one or two sentences explaining the unique contribution of this research compared with previous studies on creativity in engineering education.

 3.The conclusion in the abstract is still too general. It should emphasize the study's contribution to both the theoretical development and practical implementation of engineering education.

 Introduction

 4.The introduction mainly explains the importance of creativity in engineering education but does not clearly identify the limitations of previous studies. The research gap should be presented more explicitly and critically.

 5.The scientific contribution of the study has not been clearly articulated. The authors should explicitly state how this research advances existing knowledge.

 6.The research objectives should be formulated as Research Questions (RQs) to provide a clearer direction for the study.

 Methodology

 7.The description of the Design-Based Research (DBR) process is insufficiently detailed. The authors should systematically explain the design cycles, revision process, module development, and the relationships among each stage of the research.

 8.Information about the research participants is incomplete. The manuscript should specify the total number of participants, participant characteristics, respondent types, and their distribution across the participating countries.

 9.The qualitative data analysis procedures are not adequately described. The authors should explain the analytical techniques employed, the coding process, data validation procedures, and strategies used to ensure the credibility and trustworthiness of the findings.

 10.The research instruments are not sufficiently described. More detailed information should be provided regarding the questionnaires, measurement indicators, and the development and validation of the instruments.

 Results

 11.The Results section is dominated by descriptions of the module content. Much of this section resembles documentation of instructional material development rather than the presentation of scientific research findings. The empirical results should be strengthened.

 12.The empirical evidence presented is still limited. The authors are encouraged to provide supporting evidence through tables, figures, and quantitative or qualitative data that substantiate the findings.

 Discussion

 13.The discussion does not sufficiently relate the findings to existing theories or previous studies. The authors should explain whether the findings support, contradict, or extend previous research.

 14.The theoretical contribution of the study is not clearly demonstrated. The authors should explain how the CEDE framework contributes to the advancement of engineering education theory.

 15.Alternative interpretations of the findings are not discussed. The manuscript should acknowledge the study's limitations and consider other factors that may have influenced the results.

 Conclusion

 16.Some conclusions are stronger than the empirical evidence supports. Statements regarding the effectiveness of the platform should be presented more cautiously, as the study did not directly measure improvements in students' learning outcomes.

 17.The practical implications of the study have not been explicitly discussed. The conclusion should clearly explain how the findings can be applied in engineering education practice.

 18.The conclusion should clearly emphasize the study's contribution to the advancement of engineering education research and theory.

 19.The references rely heavily on older literature. The authors are encouraged to incorporate more recent and relevant references published between 2023 and 2026 to strengthen the theoretical foundation and demonstrate the study's relevance to current research.

Is the study design appropriate and does the work have academic merit?

Yes

Is the work clearly and accurately presented and does it cite the current literature?

Partly

If applicable, is the statistical analysis and its interpretation appropriate?

Partly

Are all the source data underlying the results available to ensure full reproducibility?

Yes

Are the conclusions drawn adequately supported by the results?

Partly

Are sufficient details of methods and analysis provided to allow replication by others?

Partly

Reviewer Expertise:

Specializing in Technical and Vocational Education and Training (TVET) and Building Engineering Education, with expertise in developing innovative learning models, educational evaluation, and strengthening the competencies of vocational teachers. Research interests include learning innovation, curriculum development, and evidence-based approaches to improving the quality of vocational education

I confirm that I have read this submission and believe that I have an appropriate level of expertise to confirm that it is of an acceptable scientific standard, however I have significant reservations, as outlined above.

# Response

**Published:** 2026-07-26

**Authors:** Cristina Prado-Acebo

**Affiliations:** Ingeniería Industrial, Universidade da Coruña, Campus Industrial de Ferrol, Escuela Universitaria de Diseño Industrial, Ferrol, La Coruña, Spain

We would like to sincerely thank the reviewer for these valuable suggestions. In response to the reviewer’s comments, we have substantially revised the manuscript to improve it.

**1-3: ** We agree that the abstract overly emphasized project activities and underrepresented the research dimension. Accordingly, the abstract has been substantially revised to:

- explicitly describe the Design-Based Research (DBR) approach;
- specify the sources of empirical data (comparative curriculum analysis and stakeholder validation round tables);
- report the main findings derived from stakeholder feedback;
- highlight the novelty of the CEDE framework as a transnational creativity-oriented model for engineering education;
- strengthen the conclusion by emphasizing both theoretical and practical contributions.

**4-6: **Thank you for the comments regarding the introduction. The introduction has been revised to more explicitly identify limitations of previous studies and to clarify the existing research gap. Specifically, we now discuss the limited availability of structured and transferable frameworks capable of integrating creativity systematically across engineering curricula and national contexts.

**7:** The DBR process has now been described in greater detail. A new subsection and figure have been incorporated, illustrating the iterative design cycles, refinement process, stakeholder feedback mechanisms and successive revisions leading to the final CEDE framework.

 8. Detailed validation data are reported for the University of A Coruña round table because this was the most complete qualitative dataset available. However, in response to the reviewer´s concern, we have now incorporated additional cross-partner evidence from Italy, Greece, Slovakia and Turkey. Since the level of detail and format of the available documentation differed across partners, these data have been analysed as a structured documentary synthesis rather than as a fully standardised comparative statistical dataset. The revised manuscript now includes a new cross-partner validation subsection and a summary table describing the stakeholder activities, participant profiles, main feedback themes and implications for the refinement of the CEDE framework.

 9. The procedure to analyse the qualitative data has now been explained.

 10. Thank you for this important observation. We agree that the original manuscript did not provide sufficient detail regarding the research instruments. To address this limitation, we have substantially expanded the Methods section by describing the questionnaire design, the assessment dimensions evaluated, the structure of the stakeholder survey, participant profiles, data collection procedures, and the process used to analyse and validate stakeholder feedback. We also clarify how the survey items were derived from the project objectives and how the instruments were reviewed by the international consortium before implementation.

 11-12. Thank you for the opportunity to highlight the scientific research findings and empirical results. The revised Results section now distinguished between the detailed UDC validation case and a broader cross-partner stakeholder validation synthesis. This addition strengthens the empirical dimension of the study by incorporating evidence from round tables and multiplier events conducted in Greece, Italy, Slovakia and Turkey, complementing the detailed qualitative finding obtained at UDC and reinforcing the transnational validation of the CEDE framework.

 13. Thank you for this valuable suggestion. We agree that the empirical evidence should be presented more explicitly. In the revised manuscript, we have substantially strengthened the Results section by distinguishing between the detailed University of A Coruña validation case and a broader cross-partner stakeholder validation synthesis. In addition to the qualitative evidence obtained through the UDC round table, we now incorporate evidence derived from round tables and multiplier events conducted by partner institutions in Greece, Italy, Slovakia and Turkey. Although the level of detail and documentation format differed across partners, the available evidence was analysed through a structured synthesis that enabled the identification of common trends across countries. The revised Results section now presents both empirical findings and stakeholder-derived recommendations in a more explicit manner. Across the participating countries, stakeholders consistently highlighted the relevance of creativity and transversal competences in engineering education, the need for more practice-oriented learning activities, the importance of robust assessment approaches, the feasibility of flexible curricular integration models, and the value of sustained university–industry collaboration. These findings are supported by stakeholder feedback obtained from students, academic staff, engineers, professional associations and company representatives involved in the validation activities. Furthermore, the empirical interpretation of these findings is framed within the Design-Based Research (DBR) approach adopted in the study. Rather than treating stakeholder feedback as a stand-alone evaluation exercise, the revised manuscript explicitly explains how this evidence informed successive cycles of refinement of the modules, assessment tools and e-learning platform. Consequently, the Results section now provides a clearer connection between empirical evidence, iterative design decisions and the final configuration of the CEDE framework.

 14. Thank you for this important observation. We agree that the theoretical contribution should be stated more explicitly. In the revised manuscript, we have strengthened the Discussion and Conclusions sections to clarify how the CEDE framework contributes to engineering education theory. Specifically, the framework extends existing research on Design Thinking, Creativity in Engineering, and Design-Based Research by proposing a transnational and transferable model that integrates creativity, critical thinking, digital thinking, artificial intelligence, problem-solving, and soft skills within a single competence-based framework. Furthermore, the study demonstrates how creativity can be systematically embedded, assessed, and validated within engineering curricula through an iterative stakeholder-centred approach. Thus, the contribution of the study goes beyond the development of educational resources and provides a conceptual framework for the design and implementation of creativity-oriented engineering education initiatives in diverse higher education contexts.

 15. Thank you for pointing that out. We have added a section on this topic: Limitations and Future Research.

 16. Thank you for this important observation. We agree that some statements in the original manuscript could be interpreted as implying educational effectiveness beyond the evidence provided. In response, we have revised the Conclusions and Discussion sections to adopt a more cautious interpretation of the findings. Specifically, claims regarding the effectiveness of the CEDE platform and modules have been reformulated to emphasize stakeholder validation, perceived relevance, feasibility, and potential educational value rather than demonstrated improvements in student learning outcomes. We also explicitly acknowledge that the study did not measure learning gains or competence development through pre- and post-intervention assessments. Consequently, the findings should be interpreted as evidence supporting the acceptability and applicability of the CEDE framework rather than its direct impact on student performance. This limitation is now clearly discussed in the manuscript, and future research directions include longitudinal evaluations of learning outcomes and competence development.

 17. Thank you for this valuable observation. We agree that the practical implications of the study should be stated more explicitly. In the revised manuscript, we have expanded the Conclusions section to clarify how the findings can be applied in engineering education practice. Specifically, we emphasise that the CEDE framework provides a transferable and flexible model that can be integrated into existing engineering curricula through complementary modules, elective courses, or embedded learning activities. The framework offers educators a structured approach for teaching and assessing creativity, critical thinking, digital thinking, problem solving, and soft skills, supported by validated learning materials, assessment tools, and an e-learning platform. Furthermore, stakeholder feedback confirmed the feasibility and relevance of these components for engineering programmes in different institutional contexts.

 18. Thank you for this important suggestion. We have revised the Conclusions and Discussion sections to highlight more clearly the study’s contribution to engineering education research and theory. From a theoretical perspective, the CEDE framework advances existing research on creativity in engineering, Design Thinking, and Design-Based Research by proposing an integrated and transnational model that conceptualises creativity as a multidimensional competence combining critical thinking, design thinking, digital thinking, problem solving, artificial intelligence, and soft skills. The study also contributes methodological knowledge by demonstrating how educational innovations can be systematically designed, refined, and validated through iterative stakeholder engagement. Consequently, the contribution of the study extends beyond the development of educational resources and provides a conceptual framework for future research and implementation of creativity-oriented approaches in engineering education.

 19. Thank you for the suggestion. We have updated the article with more recent and relevant references as indicated.

# Reviewer report: Reviewer response for version 1

**DOI:** 10.21956/openreseurope.25144.r75252

**Published:** 2026-06-12

**Authors:** Sahin Idil

**Affiliations:** 1 The Scientific and Technological Research Council of Türkiye (TÜBİTAK), Ankara, Turkey

The study focuses on five thematic areas: critical thinking, design thinking, digital problem solving, digitalisation and artificial intelligence, and soft skills. It also reports on a comparative curriculum analysis and stakeholder feedback collected through round tables involving students, academic staff, professionals, and company representatives.

 It is thought that the article addresses a timely and relevant topic. The integration of creativity, digital competencies, critical thinking, problem-solving, and soft skills into engineering curricula is highly important for contemporary engineering education. The international and collaborative nature of the CEDE project is a clear strength, and the development of concrete educational modules and an e-learning platform gives the work practical and pedagogical value. The manuscript is generally well organised and offers a useful contribution to discussions on curriculum innovation, transversal competences, and digitally supported learning environments in higher education.

 The work is presented in general terms and cites relevant literature on creativity, design thinking, engineering education, digitalisation, artificial intelligence, and transversal competences. The study also has academic merit, particularly as a project-based and developmental contribution to engineering education. The qualitative and design-based orientation of the study is appropriate for the aims of developing, refining, and validating educational modules and an e-learning platform.

 At the same time, several aspects could be clarified and strengthened to improve the article's scientific transparency, methodological robustness, and overall contribution.

 First, the research design could be described more explicitly. The authors state that the project follows a qualitative and design-based research approach, which is suitable for the purpose of the study. However, the manuscript would benefit from a clearer explanation of the phases of the study, the data sources used in each phase, the participants involved, and the way in which evidence from the comparative analysis and stakeholder feedback informed the development or refinement of the modules and the platform. More explicit alignment between the research design, data sources, analysis procedures, and reported findings would strengthen the scientific structure of the manuscript.

 Second, the comparative curriculum analysis could be presented in greater methodological detail. It would be useful to specify how many engineering programmes or curricula were analysed in each country or partner institution, what criteria were included in the common analysis template, how the data were collected and compared, and how the conclusions from this analysis were derived. At present, this part of the article is informative but relatively general. Providing more detail would increase the transparency, transferability, and potential replicability of the study.

 Third, the stakeholder feedback section is valuable, but it could be made more systematic. The manuscript states that round tables were organised in the five participating countries, while the detailed results presented mainly refer to the University of A Coruña case. If the results from the other countries were similar, this should be supported with evidence, such as a cross-country synthesis, participant numbers for each country, comparable response patterns, or a summary table. Alternatively, the authors may clarify that the detailed evidence reported mainly reflects one institutional context and discuss this as a limitation. This would make the interpretation of the stakeholder validation more precise.

 Fourth, the evaluation of the e-learning platform could be supported with additional evidence or presented more cautiously. The platform is an important output of the CEDE project, and the article describes it as useful and pedagogically valuable. However, the empirical evidence supporting the platform’s usability, effectiveness, or pedagogical impact appears limited. If usability data, student engagement data, learning analytics, platform testing results, or structured feedback specifically focused on the platform are available, they should be included. If such data are not yet available, the platform evaluation should be framed as preliminary and developmental rather than as evidence of effectiveness.

 Fifth, the conclusions could be more closely aligned with the evidence presented. The project provides a promising and structured framework for integrating creativity-related competences into engineering education. However, the current evidence mainly concerns curriculum analysis, module development, and stakeholder validation. Therefore, claims about the demonstrated effectiveness of the modules or the development of student competences should be expressed carefully. Phrases such as “the findings suggest”, “the project provides preliminary evidence”, or “the framework may offer a feasible approach” would be more appropriate in some parts of the conclusion and discussion.

 Sixth, the manuscript would benefit from minor language editing and a more consistently academic tone. Some sections, especially the descriptions of the modules, are highly descriptive and occasionally read more like a project report than a research article. These sections could be made more concise and analytical by linking the module descriptions more directly to the research aims, design process, stakeholder feedback, and findings. In addition, some broad or repetitive formulations could be revised to improve academic precision and reduce generality.

 Regarding source data and reproducibility, the manuscript should more clearly indicate which datasets, instruments, templates, questionnaires, module materials, and feedback summaries are available and where they can be accessed. Since the study relies on curriculum analysis, module design, and stakeholder feedback, the availability of supporting materials is important for transparency and reproducibility. If some data cannot be shared due to privacy or institutional constraints, this should also be clearly explained.

 The statistical analysis question is not applicable, as the study is primarily qualitative, design-based, and developmental rather than based on statistical testing.

 In summary, I consider the article to be relevant, well-motivated, and valuable for the field of engineering education. Its main strengths are the international collaboration, the timely focus on creativity and transversal competences, and the practical development of educational modules and an e-learning platform. I recommend that the authors revise the manuscript by clarifying the research design, strengthening the methodological description, providing more systematic evidence from the stakeholder feedback, presenting the platform evaluation either with stronger evidence or with more cautious wording, and aligning the conclusions more closely with the data. These revisions would considerably strengthen the article’s scientific transparency, methodological clarity, and contribution to the field.

Is the study design appropriate and does the work have academic merit?

Yes

Is the work clearly and accurately presented and does it cite the current literature?

Yes

If applicable, is the statistical analysis and its interpretation appropriate?

Not applicable

Are all the source data underlying the results available to ensure full reproducibility?

Partly

Are the conclusions drawn adequately supported by the results?

Partly

Are sufficient details of methods and analysis provided to allow replication by others?

Partly

Reviewer Expertise:

My areas of expertise include STEM education, digital education, artificial intelligence in education, curriculum development, design thinking, project-based learning, and the development of transversal competences such as creativity, critical thinking, problem solving, collaboration, and communication. I am able to assess the article particularly in terms of its educational design, methodological clarity, curriculum relevance, e-learning approach, and contribution to engineering education.

I confirm that I have read this submission and believe that I have an appropriate level of expertise to confirm that it is of an acceptable scientific standard, however I have significant reservations, as outlined above.

# Response

**Published:** 2026-06-16

**Authors:** Cristina Prado-Acebo

**Affiliations:** Ingeniería Industrial, Universidade da Coruña, Campus Industrial de Ferrol, Escuela Universitaria de Diseño Industrial, Ferrol, La Coruña, Spain

We would like to sincerely thank the reviewer for their thorough and constructive assessment of our manuscript, as well as for recognising the relevance and potential contribution of the CEDE project to engineering education. In response to the reviewer’s comments, we have substantially revised the manuscript to improve its scientific transparency, methodological clarity, and alignment between evidence and conclusions.

1. First, we have strengthened the description of the research design by explicitly structuring the study according to a design-based research approach, clarifying its phases, data sources, participant groups, and the relationships between analysis, design, and evaluation processes.
2. Second, the methodological detail of the comparative curriculum analysis has been expanded, including a clearer description of the analysis template, criteria, data collection procedures, and cross-institutional comparison process.
3. Third, the stakeholder feedback section has been revised to provide a more systematic and transparent account. We have clarified the multi-country nature of the round tables, included a cross-institutional perspective, and explicitly acknowledged the level of detail available for specific case studies.
4. Fourth, we have carefully revised the sections related to the e-learning platform and module descriptions to adopt a more analytical and research-oriented tone. In line with the reviewer’s suggestions, claims about effectiveness have been moderated, and the platform is now presented as a developmental and iterative outcome supported by initial feedback.
5. Fifth, the discussion and conclusions have been substantially refined to ensure that all claims are fully supported by the available evidence. We have adopted more cautious and precise formulations, emphasising that the findings provide preliminary evidence and a structured framework rather than definitive proof of effectiveness.
6. Finally, the manuscript has undergone language editing and restructuring to improve clarity, reduce redundancy, and ensure a consistent academic tone throughout. Additional information has also been included regarding data availability and supporting materials to enhance transparency and reproducibility. We believe that these revisions have significantly strengthened the manuscript and we are grateful to the reviewer for their valuable suggestions.
